# Photoreceptor-induced RPE phagolysosomal maturation defects in Stargardt-like Maculopathy (STGD3)

**DOI:** 10.1038/s41598-018-24357-4

**Published:** 2018-04-13

**Authors:** Camille Dejos, Sharee Kuny, Woo Hyun Han, Heather Capel, Hélène Lemieux, Yves Sauvé

**Affiliations:** 1 Department of Ophthalmology and Visual Sciences, University of Alberta, 7-45 Medical Sciences Building, Edmonton, AB T6G 2H7 Canada; 2 Department of Physiology, University of Alberta, 7-45 Medical Sciences Building, Edmonton, AB T6G 2H7 Canada; 3 Faculty Saint-Jean, University of Alberta, 8406 Rue Marie-Anne Gaboury Northwest, Edmonton, AB T6C 4G9 Canada

## Abstract

For many neurodegenerative disorders, expression of a pathological protein by one cell type impedes function of other cell types, which in turn contributes to the death of the first cell type. In transgenic mice modelling Stargardt-like (STGD3) maculopathy, human mutant ELOVL4 expression by photoreceptors is associated with defects in the underlying retinal pigment epithelium (RPE). To examine how photoreceptors exert cytotoxic effects on RPE cells, transgenic ELOVL4 (TG1–2 line; TG) and wild-type (WT) littermates were studied one month prior (preclinical stage) to onset of photoreceptor loss (two months). TG photoreceptor outer segments presented to human RPE cells are recognized and internalized into phagosomes, but their digestion is delayed. Live RPE cell imaging pinpoints decreased numbers of acidified phagolysomes. *In vivo*, master regulator of lysosomal genes, transcription factor EB (TFEB), and key lysosomal enzyme Cathepsin D are both unaffected. Oxidative stress, as ruled out with high-resolution respirometry, does not play a role at such an early stage. Upregulation of CRYBA1/A3 and phagocytic cells (microglia/macrophages) interposed between RPE and photoreceptors support adaptive responses to processing delays. Impaired phagolysosomal maturation is observed in RPE of mice expressing human mutant ELOVL4 in their photoreceptors prior to photoreceptor death and associated vision loss.

## Introduction

Mutations in *ELOVL4*, responsible for a dominant maculopathy (Stargardt-like, STGD3), lead to an enzymatically inactive truncated protein lacking its C-terminal endoplasmic reticulum retention signal^1^. Unfolded protein response (UPR) alone should initiate death in cells expressing the truncated ELOVL4 protein, as occurs in cell lines (COS-7 and HEK-293 cells)^2^. However, transgenic mice expressing mutant ELOVL4 in their photoreceptors do not display evidence of UPR until advanced cell death^3,4^. Furthermore, loss of ELOVL4 elongase activity^5^, essential for the synthesis of very long chain polyunsaturated fatty acids (VLC-PUFAs, of 28–36 carbon chains)^1^, does not cause retinal degeneration in STGD3^3,6^.

The mechanisms initiating retinal degeneration in STGD3 still elude us and are the subject of intense debate. Human mutant ELOVL4 mislocalizes to outer segments when expressed in the photoreceptors of *Xenopus laevis*^7^; whether it also mislocalizes to the much thinner outer segments of mice has yet to be elucidated. A major hurdle is the inability to generate an antibody specific to the truncated ELOVL4 protein. Furthermore, while ELOVL4 transgenic mice have been studied in detail, this model does not express a tag on the transgene. We previously described that transgene expression in photoreceptors leads to perturbations in disc membrane ultrastructure and signs of RPE cell toxicity, prior to cell death^8^. A clinical hallmark of STGD3 is the accumulation of a toxin, lipofuscin (a non-enzymatic by-product of vitamin A aldehyde), formed in photoreceptors and transferred to RPE cells through daily outer segment phagocytosis, which exerts deleterious effects on the RPE^9^.

One hypothesis receiving increasing scrutiny is that for cells expressing a mutant protein to die, these cells must first alter the physiology of other cell types^10^. Survival requirements of highly specialized neurons involve interactions with supporting cells. Photoreceptor survival depends on the daily shedding and degradation of their outer segments (POS) by adjacent RPE cells^11^. Sequential steps including recognition, ingestion and degradation, are involved. Defects in any of these cellular processes lead to photoreceptor death. Impaired outer segment tip recognition leads to the accumulation of lipofuscin in the RPE and of toxic debris in the subretinal space^12^. Defects in ingestion, cause dry AMD-like phenotypes^13^. Finally, defective lysosomal degradation of POS content, as reported in mice lacking Cathepsin D^14^ or expressing mutant beta-crystallin A1/A3^15^, is associated with increased lipofuscin levels, which have been reported to impede lysosomal function in AMD^16^. In Stargardt hereditary maculopathies, and the more common age-related macular degeneration that affects close to 200 million worldwide^17^, photoreceptor death and vision loss is preceded by asymptomatic RPE defects.

Previous findings in the transgenic ELOVL4 (TG1–2) mouse showed signs of RPE toxicity *in vivo* (disorganization of apical villi and vacuolization) at one month of age, followed at 2 months by photoreceptor cell death onset, and at 3 months by phagocytic defects (delayed phagosome movement, undigested outer segments and lipid deposits)^8^ and increased A2E levels^18^. This study examines how RPE cells are affected by the expression of mutant ELOVL4 in photoreceptors, prior to cell death.

## Results

### Processing of POS isolated from TG mice is delayed *in vitro*

Since the RPE of TG mice (with photoreceptoral expression of human truncated ELOVL4 protein) already demonstrates evidence of toxicity^8^, we hypothesize that POS presentation leads to lysosomal dysfunction in the RPE. To examine the cytotoxic effect of TG-POS on RPE cells, we presented isolated POS from TG and WT littermate mice to human RPE *in vitro*.

**Figure 1:**
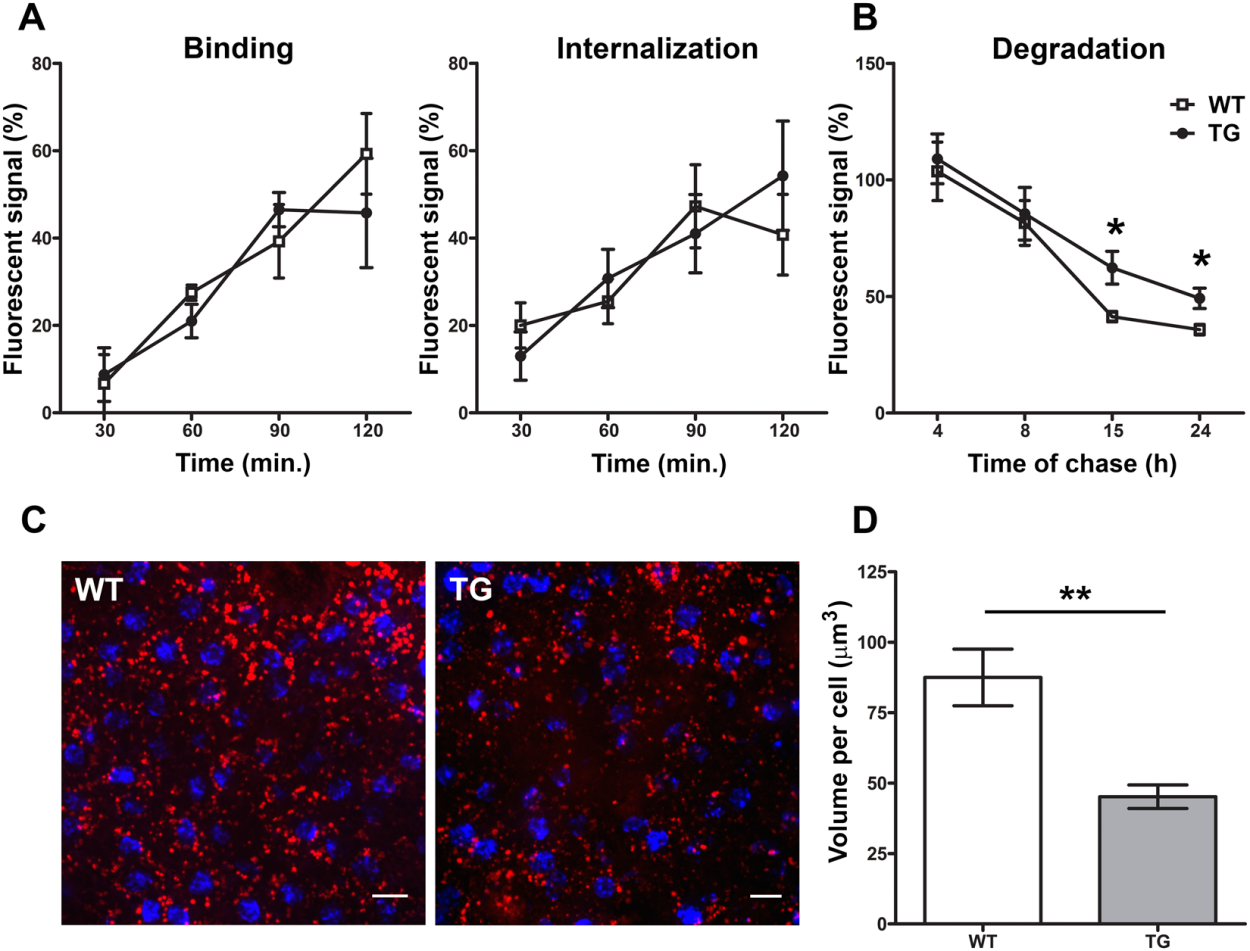
Degradation of TG POS is delayed. Numbers of phagolysosomes are reduced. *In vitro*: (**A**) Graphs representing the kinetics of binding (OS bound but not internalized) and internalization (OS bound and internalized). Human RPE were presented with Alexa Fluor 488-labeled POS, isolated from WT (white squares) or TG (black circles) mice, for 120 minutes. Kinetics of POS binding and internalization were calculated using total fluorescence and internal signal from phagosomes after quenching. Data are expressed as mean ± SEM; n = 3–4 independent experiments. (**B**) Graph showing phagocytic pulse-chase assay. Human RPE were presented with POS for 2 hours, and fluorescence levels were quantified at different times following POS removal. Fusion of phagosomes containing fluorescently-labelled POS with acidified lysosomes, leads to POS degradation and loss of fluorescence signal, expressed here on the y axis as percentage of signal at time zero of presentation. Data are expressed as mean ± SEM; n = 3–5 independent experiments. *P* ≤ *0.05. Live cell imaging: (**C**) Representative confocal images of acidified phagolysosomes labelled with pH sensitive dye (red) and nuclei stained with DAPI (blue) on freshly dissected RPE flatmounts, from WT (left) and TG (right) mice. Animals were culled 2 hours after light onset (7AM). (Scale bar: 10 μm). (**D**) Histogram showing the volume of acidified phagolysosomes occupied inside single RPE cells. Data are expressed as mean ± SEM; n = 13 microscope fields per group. **P ≤ 0.005.

The kinetics of three distinct phagocytic steps was studied: POS binding, internalization into phagosomes and lysosomal degradation. Human RPE cells were challenged with fluorescently labelled POS over a 2-hour time period. A progressive increase in fluorescence signal demonstrates that RPE cells bind and internalize POS isolated from TG and WT mice with the same kinetics (Fig. 1A). Therefore, RPE cells have the ability to recognize POS from TG mice, form phagosomes and internalize them.

We then quantified the ability of RPE cells to digest POS over time by performing a 2 hour pulse period with POS, followed by chase periods of increasing duration (Fig. 1B). After a 4 hour chase period, the amount of fluorescence is still comparable to initial levels measured after 2 hours incubation with POS. From 4 to 8 hours, the fluorescence signal decreases at the same rate for cells incubated with either TG or WT POS (1.3-fold decrease). However, between 8 and 15 hours, fluorescence decreases at a slower rate in the group presented with TG-POS (1.4-fold vs 2.0-fold decrease). This gap persists after 24 hours of chase with the percentage of fluorescent signal remaining higher for RPE cells digesting TG-POS (49.4 ± 4.39 vs 35.7 ± 2.23; P = 0.0278). This slower decrease in fluorescence suggests that phagosome fusion with lysosomes is impeded when phagosomes contain TG-POS.

### Acidified phagolysosome maturation is impaired

We quantified phagolysosomes using live cell imaging of RPE in the presence of a pH-sensitive acidophilic fluorophore^19^ at 2 hours after light onset (Fig. 1C), during the predicted burst of POS uptake. In TG mice, acidified phagolysosomes occupy a 2.0-fold smaller total volume within RPE cells (Fig. 1D, P = 0.001). This difference is due to a lower number of phagolysosomes per cell (17 ± 1 in TG vs 28 ± 3 in WT; P = 0.001; n = 13 images per group) and not to a difference in phagosome volume (1.77 ± 1.01 µm^3^ vs 1.84 ± 1.03 µm^3^). The reduced abundance of acidified phagolysosomes in RPE cells reflects a maturation defect that could either be due to decreased fusion of phagosomes with lysosomes and/or to impaired acidification.

### Preserved activation of autophagy-lysosomal pathway components

**Figure 2:**
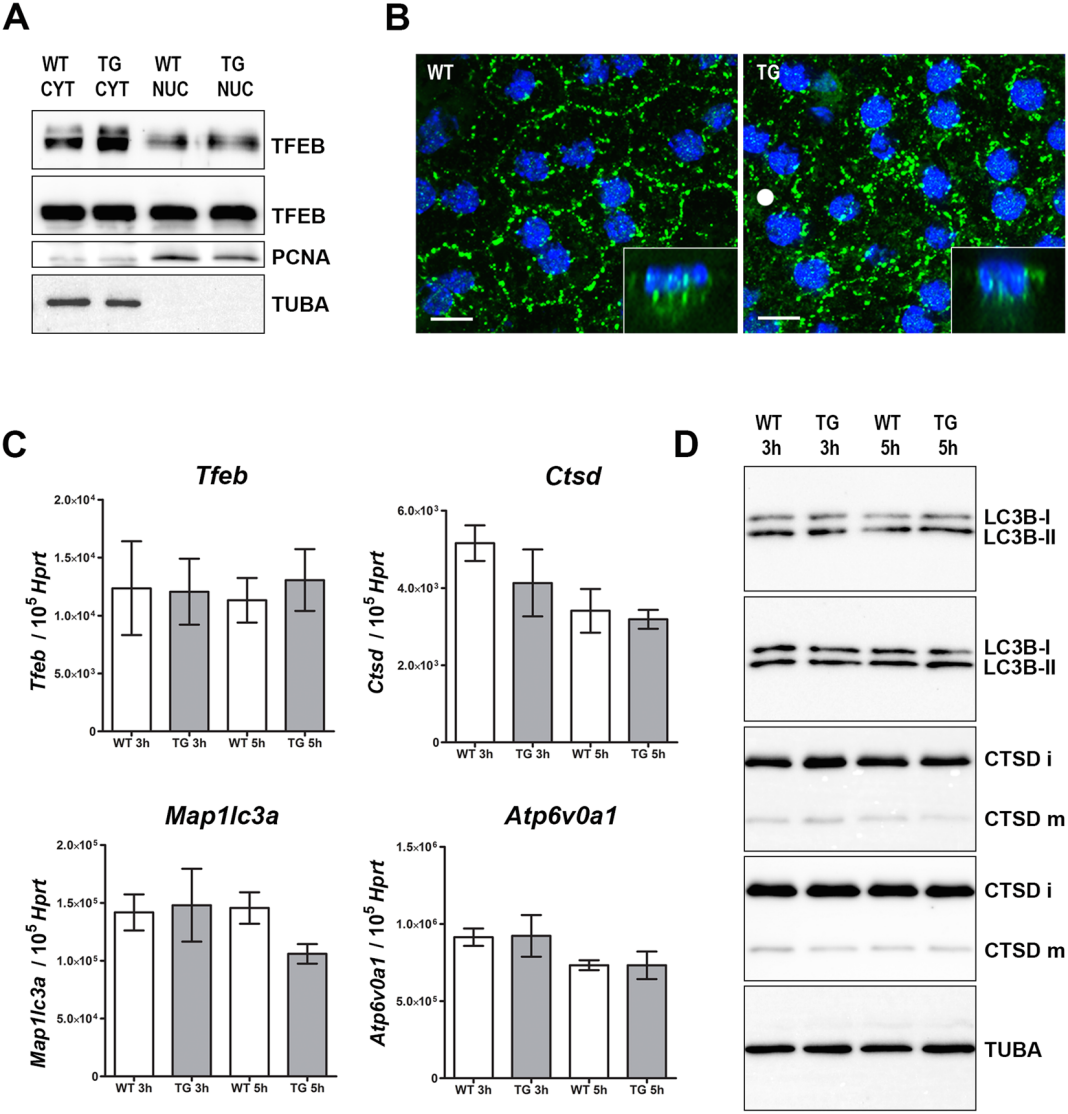
Autophagy-lysosomal pathway genes and proteins have normal expression levels. (**A**) Immunoblots of TFEB, PCNA and TUBA using cytoplasmic proteins (5 µg per lane) and nuclear proteins (2 µg per lane) from RPE homogenates prepared 2 hours after light onset (7AM). (**B**) Representatives confocal images of TFEB (green) and DAPI (blue) immunochemistry in WT (left) and TG (right) mice (Scale bar: 10 µm). (**C**) Histogram showing relative *Tfeb*, *Map1lc3a*, *Ctsd* and *Atp6v0a1* mRNA levels in the RPE by qRT-PCR. Each mRNA level was normalized to *Hprt*. Total RNA was prepared 3 hours (8AM) and 5 hours (10AM) after light onset. Data represent mean ± SEM; n = 5–8 mice per group. (**D**) Immunoblots of LC3B-I/LC3B-II, CTSD (i, immature and m, mature) and TUBA using RPE homogenates prepared 3 hours (8AM) and 5 hours (10AM) after light onset.

Phagocytosis of OS by RPE cells induces activation of Transcription Factor EB (TFEB)^20^, a master gene for lysosomal biogenesis^21^ and an effector of lysosomal function when translocated to the nucleus^22^. We quantified with western blot the respective cytoplasmic and nuclear fractions of TFEB. The ratio of cytoplasmic over nuclear TFEB (examined at 2 hours after light onset) is equivalent in TG and WT mice (Fig. 2A, means of 0.50 vs 0.46, from two independent western blots). TFEB nuclear translocation is occurring at normal levels. Characterization of the cellular location of TFEB by immunofluorescence confirms the presence of TFEB in the nuclei of both TG and WT RPE (Fig. 2B). In TG, TFEB-labelled vesicles are diffusely distributed within the RPE cytosol. Since TFEB acts as a sensor of lysosomal state when located on the lysosomal surface^22^, these findings warrant further studies.

Next we evaluated the transcriptional activation of *Tfeb* and of select targets of the Coordinated Lysosomal Expression and Regulation (CLEAR) gene network^23^. *Tfeb* is expressed at WT levels in the hours following POS engulfment (Fig. 2C). Downstream CLEAR genes *Map1lc3a*, *Ctsd* and *Atp6v0a1* are similarly unaffected. Finally, we verified unchanged protein levels of microtubule-associated protein 1 light chain 3 beta (LC3B) and cathepsin D (CTSD) (Fig. 2D).

### Members of the crystallin protein family are upregulated in RPE

**Figure 3:**
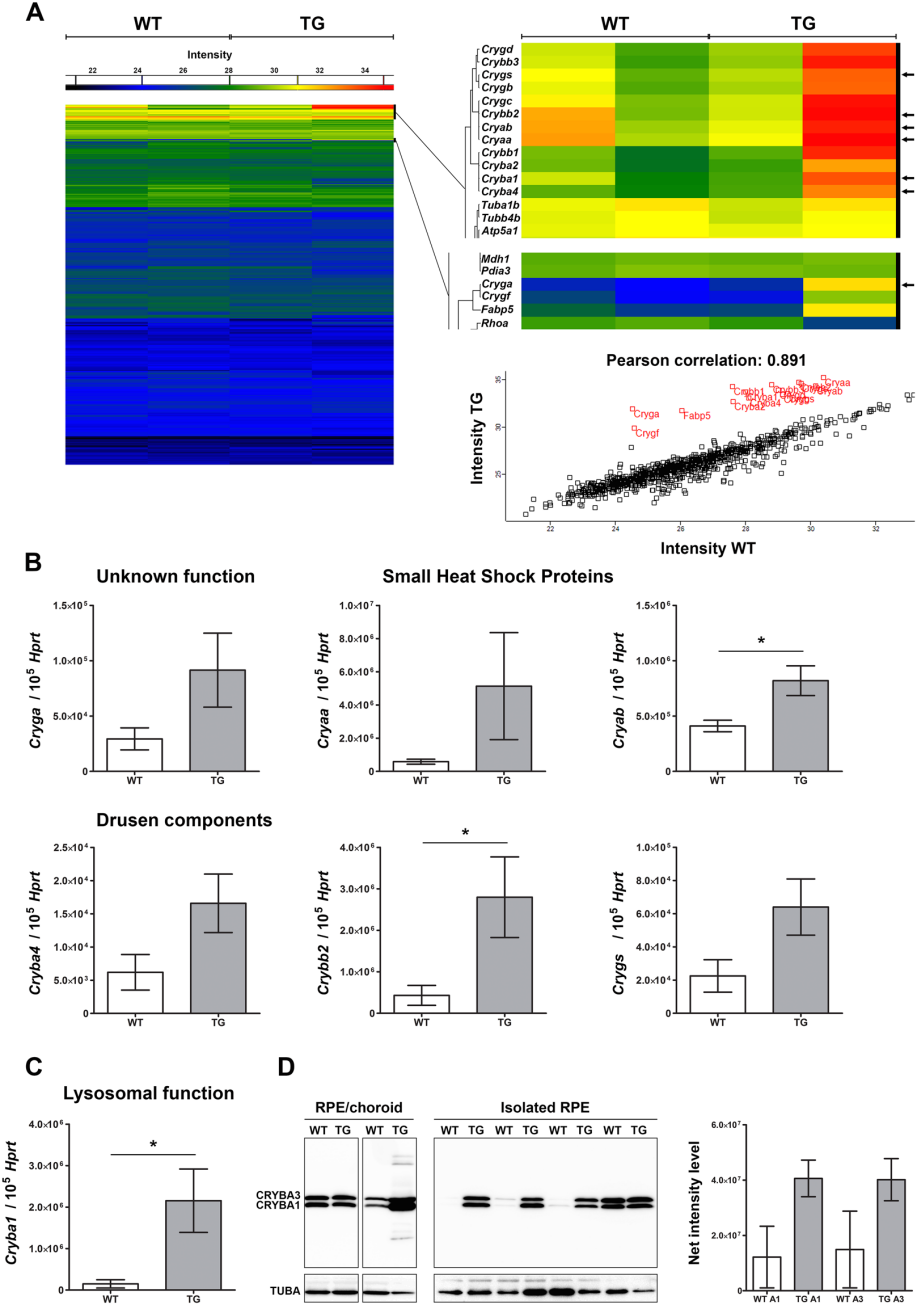
Crystallin genes and proteins are differentially expressed. (**A**) Heatmap showing expression levels of 963 proteins detected in RPE/choroid homogenates from WT (left) and TG (right) mice (2 independent experiments). Eyecup pool WT, n = 4 mice; eyecup pool TG n = 3–4 mice. Zoom showing relative intensities for the crystallin protein cluster. Expression of genes coding for targeted crystallin family members was further evaluated by qRT-PCR (black arrows). Graph showing Pearson correlation (2 independent experiments). (**B**) Histogram showing relative mRNA levels of drusen components (*Cryba4*, *Crybb2* and *Crygs*), genes coding for small heat shock proteins (*Cryaa* and *Cryab*) and *Cryga* in the RPE by qRT-PCR. Each mRNA level was normalized to *Hprt*. Data represent mean ± SEM; n = 6–10 mice per group. *P ≤ 0.05. (**C**) Histogram showing relative mRNA levels of *Cryba1*, involved in lysosomal function, in the RPE by qRT-PCR. Each mRNA level was normalized to *Hprt*. Data represent mean ± SEM; n = 7–9 mice per group. *P ≤ 0.05. (**D**) Immunoblots of CRYBA1/3 and TUBA using RPE/choroid homogenates analysed by mass spectrometry (left, proteins from 3–4 mice per lane) and isolated RPE protein extracts (right, 1 animal per lane, n = 4 each group).

In an attempt to identify the signaling pathways affected in TG mice following POS phagocytosis, we conducted RPE proteomics. This analysis demonstrates that not all TG mice are showing changes at this early stage, as highlighted in the proteomic heatmap (Fig. 3A). Across all samples, 963 proteins were analysed and compared. The close proximity of both proteomes is reflected by a Pearson correlation coefficient of 0.891, obtained when averaged protein abundances are plotted against each other (Fig. 3A). Table 1 further highlights variations in protein expression between duplicates of pooled TG eyecups. Only 10 proteins are differentially expressed in a range of 4.13 to 0.11 fold-change TG pool A. Whereas TG pool B showed 64 proteins with differential expression levels and fold-change ranging from 122.32 to 0.07. One major cluster demonstrating overexpression includes the alpha-, beta- and gamma-crystallin family members (Fig. 3A; Table 1).

**Table 1:** Variation in Protein Levels in TG eyecups at one month. Threshold is set at 3-fold change.

Uniprot ID	Gene name	Protein Name	Fold Change/WT
**TG eyecup pool A**			
A2AEN9	*Gm5938*	Uncharacterized protein	4.13
Q02257	*Jup*	Junction plakoglobin	3.78
Q9CYZ2	*Tpd52l2*	Tumor protein D54	3.41
A2BHD2	*Gm14743*	Uncharacterized protein	3.24
Q00898	*Serpina1e*	Alpha-1-antitrypsin 1-5	3.03
Q91Z53	*Grhpr*	Glyoxylate/hydroxypyruvate reductase	0.33
P30999	*Ctnnd1*	Catenin delta-1	0.31
Q9Z1Z0	*Uso1*	General vesicular transport factor p115	0.30
O35639	*Anxa3*	Annexin A3	0.29
P09470	*Ace*	Angiotensin-converting enzyme	0.11
**TG eyecup pool B**			
P04345	*Cryga*	Gamma-crystallin A	122.32
Q9WVJ5	*Crybb1*	Beta-crystallin B1B	52.31
Q05816	*Fabp5*	Fatty acid-binding protein	35.50
Q9JJU9	*Crybb3*	Beta-crystallin B3	24.54
Q9CXV3	*Crygf*	Gamma-crystallin F	21.58
Q9JJV0	*Cryba4*	Beta-crystallin A4	21.35
P02525	*Cryba1*	Beta-crystallin A1	20.06
Q9JJV1	*Cryba2*	Beta-crystallin A2	17.75
P04342	*Crygd*	Gamma-crystallin D	16.54
Q61597	*Crygc*	Gamma-crystallin C	15.33
P24622	*Cryaa*	Alpha-crystallin A chain	12.82
P62696	*Crybb2*	Beta-crystallin B2	12.37
P04344	*Crygb*	Gamma-crystallin B	10.04
O35486	*Crygs*	Gamma-crystallin S	8.93
P23927	*Cryab*	Alpha-crystallin B chain	7.91
Q8BKC5	*Ipo5*	Importin-5	6.12
Q78ZA7	*Nap1l4*	Nucleosome assembly protein 1-like 4	4.62
Q99K85	*Psat1*	Phosphoserine aminotransferase	4.51
Q921M7	*Fam49b*	Protein FAM49B	3.82
P16330	*Cnp*	2,3-cyclic-nucleotide 3-phosphodiesterase	3.77
P14602	*Hspb1*	Heat shock protein beta-1	3.60
P53994	*Rab2a*	Ras-related protein Rab-2A	0.33
P97457	*Mylpf*	Myosin regulatory light chain 2	0.33
P22599	*Serpina1b*	Alpha-1-antitrypsin 1-2	0.32
Q9WVA4	*Tagln2*	Transgelin-2	0.32
P35980	*Rpl18*	60S ribosomal protein L18	0.32
P62874	*Gnb1*	Guanine nucleotide-binding protein G(I)/G(S)/G(T) subunit beta-1	0.32
O35643	*Ap1b1*	AP-1 complex subunit beta-1	0.31
P14115	*Rpl27a*	60S ribosomal protein L27a	0.31
P48774	*Gstm5*	Glutathione S-transferase Mu 5	0.31
P30412	*Ppic*	Peptidyl-prolyl cis-trans isomerase C	0.30
Q9DB20	*Atp5o*	ATP synthase subunit O	0.29
F6YVP7	*Gm10260*	40S ribosomal protein S18	0.29
Q8VEH3	*Arl8a*	ADP-ribosylation factor-like protein 8A	0.28
Q99JI6	*Rap1b*	Ras-related protein Rap-1b	0.28
P62717	*Rpl18a*	60S ribosomal protein L18a	0.28
Q60692	*Psmb6*	Proteasome subunit beta type-6	0.28
Q9QZ88	*Vps29*	Vacuolar protein sorting-associated protein 29	0.27
Q9DCZ4	*Apoo*	Apolipoprotein O	0.27
Q9CYH2	*Fam213a*	Redox-regulatory protein FAM213A	0.27
Q9CQC9	*Sar1b*	GTP-binding protein SAR1b	0.26
O09167	*Rpl21*	60S ribosomal protein L21	0.25
Q62000	*Ogn*	Mimecan	0.24
Q8BL97	*Srsf7*	Serine/arginine-rich splicing factor 7	0.23
Q61171	*Prdx2*	Peroxiredoxin-2	0.22
Q99NB9	*Sf3b1*	Splicing factor 3B subunit 1	0.22
P09528	*Fth1*	Ferritin heavy chain	0.20
Q9QZ47	*Tnnt3*	Troponin T	0.19
Q9CY50	*Ssr1*	Translocon-associated protein subunit alpha	0.19
P35282	*Rab21*	Ras-related protein Rab-21	0.19
P35278	*Rab5c*	Ras-related protein Rab-5C	0.18
P97461	*Rps5*	40S ribosomal protein S5	0.18
P60766	*Cdc42*	Cell division control protein 42 homolog	0.17
P62835	*Rap1a*	Ras-related protein Rap-1A	0.16
P35293	*Rab18*	Ras-related protein Rab-18	0.16
Q9CPR4	*Rpl17*	60S ribosomal protein L17	0.16
Q9QUI0	*Rhoa*	Transforming protein RhoA	0.15
F6QL70	*Gm17669*	60S ribosomal protein L29	0.14
O08599	*Stxbp1*	Syntaxin-binding protein 1	0.14
P84104	*Srsf3*	Serine/arginine-rich splicing factor 3	0.14
P09470	*Ace*	Angiotensin-converting enzyme	0.13
O08547	*Sec. 22b*	Vesicle-trafficking protein SEC. 22b	0.13
P97315	*Csrp1*	Cysteine and glycine-rich protein 1	0.09
P63001	*Rac1-2-3*	Ras-related C3 botulinum toxin substrate 1-2-3	0.07

Targeted crystallin family members (Fig. 3A arrows) were examined in further detail. The greatest increase in crystallin protein expression is for CRYGA (122.32-fold). However, examination of gene expression in individual animals reveals preserved mRNA levels (Fig. 3B; P = 0.113). The level of *Cryaa* is similarly unchanged (small heat shock protein; P = 0.201)^24^. *Cryab* gene expression was only increased by 2.0-fold (P = 0.022). Whether this borderline upregulation might reflect a neuroprotective response to oxidative stress^25^ is unlikely in view of our respirometry results (see below). CRYBA4, CRYBB2, and CRYGS, are found in drusen deposits^26^. Expression levels support their upregulation in TG mice; however, only the *Crybb2* increase (6.5-fold) is statistically significant (P = 0.040). Subretinal deposits are not seen in TG mice until after cell death onset^8^.

We quantified mRNA and protein levels of beta-crystallin A1/A3, a regulator of lysosomal function^15^. *Cryba1* expression is 14.4-fold higher in TG (P = 0.031; Fig. 3C). Western blot analysis of the RPE/choroid protein samples used for proteomics (Fig. 3D) confirmed that CRYBA1/A3 levels in TG can either be equal to WT littermates or elevated. To further evaluate CRYBA1/A3 expression levels we prepared and analysed RPE-enriched proteins isolated from single animals. CRYBA1/A3 expression levels remain consistently high in TG mice. In contrast, WT mice display a wide range of expression levels, indicative of tightly regulated CRYBA1/A3.

### RPE cells conserve their mitochondrial respiration capacity and show no sign of oxidative stress

Since the RPE is such a metabolically active tissue, it is particularly vulnerable to oxidative stress. Mitochondria are both a source of oxidative stress and a highly vulnerable target for oxidative damage. For this reason, we examined mitochondrial oxidative phosphorylation (OXPHOS) capacity in RPE using high-resolution respirometry (Oxygraph 2k; Oroboros), as a sensitive indicator of early oxidative stress in the RPE.

**Figure 4:**
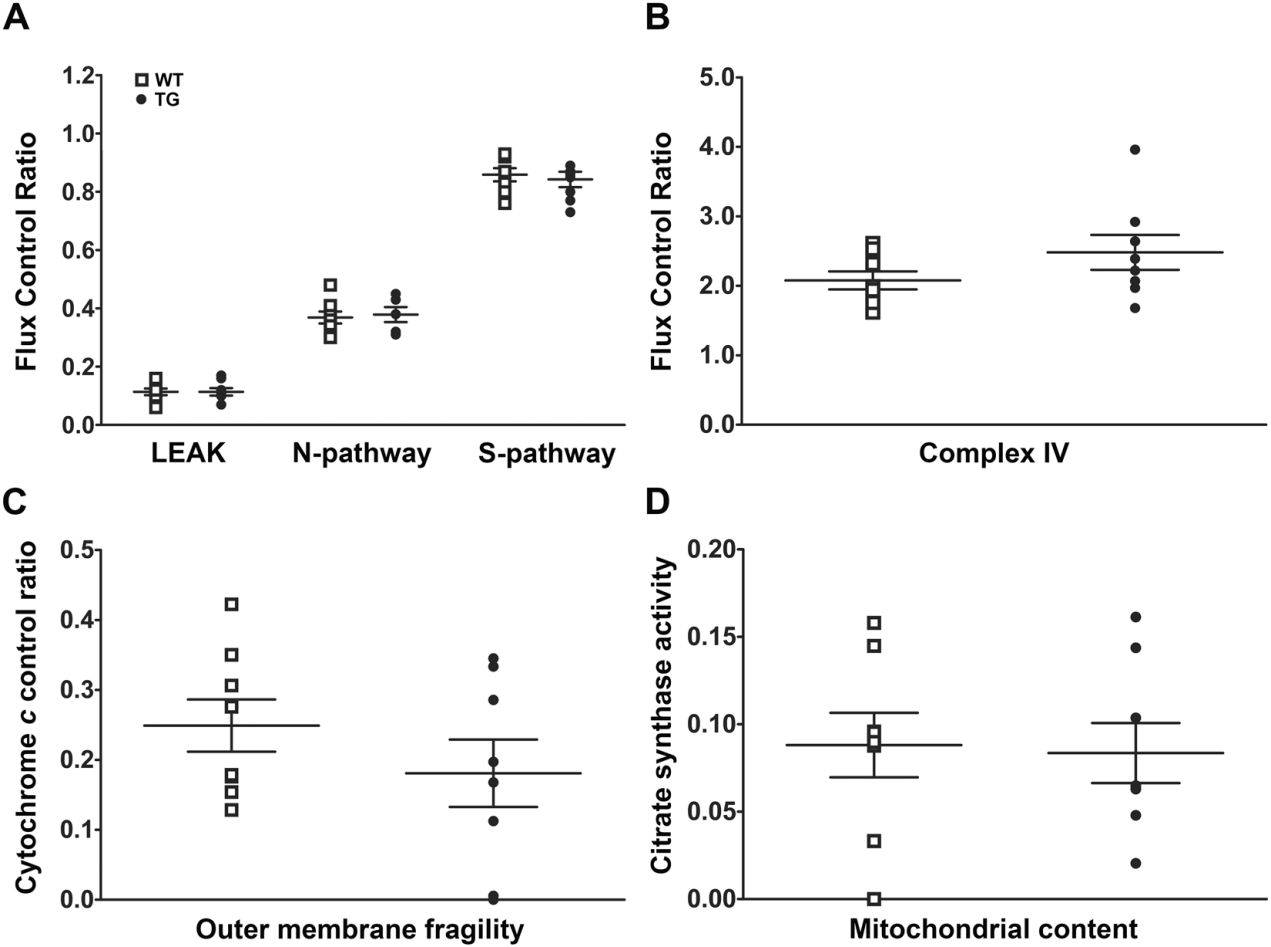
Mitochondrial oxidative phosphorylation and mitochondrial content are preserved. (**A**) Dot plot of flux control ratios for LEAK, NADH (N)- and Succinate (S)- OXPHOS pathways. (**B**) Dot plot presenting flux control ratios for Complex IV single step. (**C**) Dot plot showing cytochrome *c* control ratio. (**D**) Dot plot of mitochondrial content as assessed by citrate synthase enzymatic activity assays. Data are presented as mean ± SEM; n = 8 independent experiments.

Well-coupled mitochondria respond to the introduction of substrates, ADP and inhibitors. Mitochondrial function was measured as (1) LEAK respiration, a non-phosphorylated state in the absence of ADP but in the presence of substrates feeding the NADH pathway (pyruvate and malate), and (2) OXPHOS capacity, oxygen consumption coupled to the phosphorylation of ADP to ATP. OXPHOS capacity was evaluated for both the NADH- (pyruvate and malate), and Succinate- (succinate and rotenone) pathways, as well as for the single step of Complex IV (CIV, cytochrome *c* oxidase). The results demonstrate no differences between groups for any of the mitochondrial functions measured. The values in fluxes per mass are as follows (in pmol/s·mg for TG vs WT animals, respectively): LEAK respiration, 0.42 (0.00–3.37) vs 0.42 (0.00–2.96), P = 0.88; OXPHOS capacity for the NADH-pathway, 5.94 (4.20–8.94) vs 6.94 (4.03–13.72), P = 0.34; Succinate-pathway, 12.67 (10.58–24.01) vs 18.67 (9.43–23.10), P = 0.23; and Complex IV, 43.07 (22.22–54.49) vs 41.10 (19.68–72.54), P = 0.89. To account for subtle qualitative differences in the OXPHOS system, respiration data were also expressed as Flux Control Ratios (FCR), normalized for maximal OXPHOS capacity in the presence of electrons feeding the NADH and the Succinate pathways simultaneously (NS-OXPHOS). The FCRs also fail to detect any significant differences between groups (Fig. 4A and B).

The addition of cytochrome *c* was used as an indicator of mitochondrial outer membrane integrity and expressed as a cytochrome *c* control factor (fractional change of respiration from the state without cytochrome *c* to the state stimulated by exogenous cytochrome *c*)^27^. A value of zero (0.00) indicates full membrane integrity (no loss of endogenous cytochrome *c*), while a value of one (1.00) signifies maximal damage. Cytochrome *c* control factors are comparable between groups 0.18 pmol/s•mg (0.00–0.35; min-max) vs 0.23 (0.13–0.42), P = 0.28 (Fig. 4C). The values presented are higher than previously obtained with retinas^28^, likely reflecting the more aggressive mechanical disruption required for homogenizing eyecups. This addition of exogenous cytochrome *c* also served to remove the bias induced by limitation of cytochrome *c* availability (as a result of sample preparation). Finally, mitochondrial content as assessed by citrate synthase activity is comparable between groups (Fig. 4D).

Preserved mitochondrial function excludes the possibility of oxidative stress induced by changes in mitochondrial oxidative phosphorylation capacity.

### Microglia/macrophages infiltrate the subretinal space

**Figure 5:**
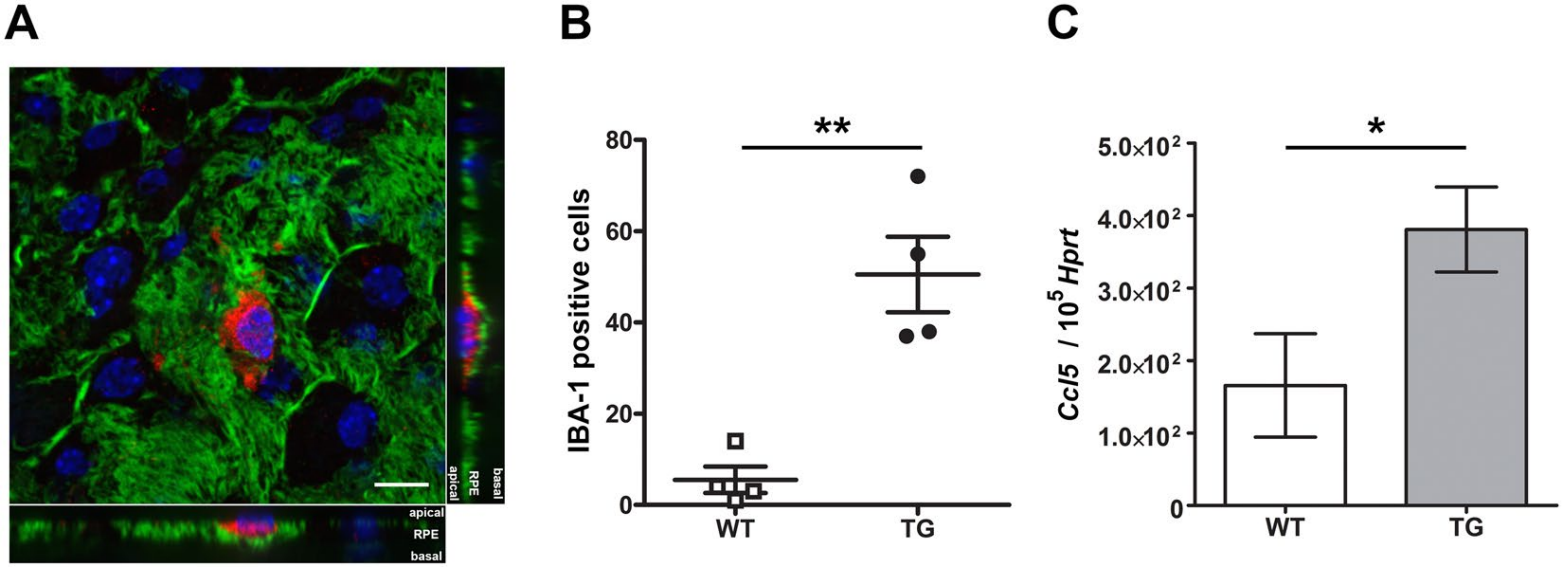
Microglia/macrophages infiltrate the subretinal space. (**A**) Confocal image of a cell reactive for IBA-1 (red), actin filaments labelled with phalloidin (green) and nuclei stained with Hoechst (blue). Z-stack orthogonal projections (right, bottom) show an IBA-1 positive cell located on the apical side of the RPE. (Scale bar: 10 μm). (**B**) Dot plot of the number of IBA-1 positive cells per RPE flatmount. Data are presented as mean ± SEM; n = 4 eyes per group. **P ≤ 0.005. (**C**) Histogram showing relative *Ccl5* mRNA levels in the retina by qRT-PCR. mRNA levels were normalized to *Hprt*. Data represent mean ± SEM; n = 7–8 mice per group. *P ≤ 0.05.

Prior to detectable subretinal debris accumulation, cells were observed on the RPE apical surface and were identified as being of the microglia/macrophage lineage (IBA-1 + labelling; Fig. 5A).

Microglia/macrophages are particularly abundant in the central part of the eye where photoreceptoral degeneration first begins in this TG model^4,29^. Confocal imaging confirms that the microglia/macrophages are at the RPE apical surface and therefore within the subretinal space (Fig. 5A). The number of IBA-1 positive cells is 8.5-fold higher in TG mice (51 ± 17 vs 6 ± 3 cells per eye; mean ± SEM; n = 4 eyes; P = 0.014; Fig. 5B).

Evaluation by qPCR (in RPE vs neural retina) for the expression of chemotactic cytokines reveals a 2.4-fold upregulation of Chemokine (C-C motif) ligand 5 (*Ccl5* or RANTES), a macrophage-activating chemokine^30^, in TG retinas (P = 0.015; Fig. 5C). Expression of *Ccl2*, a leukocyte-attracting signal released from the RPE/choroid with age, is low in both TG and WT mice (does not reach minimal signal intensity).

## Discussion

Despite detailed scrutiny, the events initiating photoreceptor death in STGD3, and many other neurodegenerative disorders, are unknown. In STGD3, photoreceptors possess the primary defect (expression of mutant ELOVL4 enzyme) and their death is responsible for vision loss. We know that loss of ELOVL4 elongase activity^5^, essential for the synthesis of very long chain polyunsaturated fatty acids (VLC-PUFAs, of 28–36 carbon chains)^1^ does not cause retinal degeneration in STGD3^3,6^. Therefore, STGD3 presents itself as a gain of function disease with mutant ELOVL4 playing a direct role.

**Figure 6:**
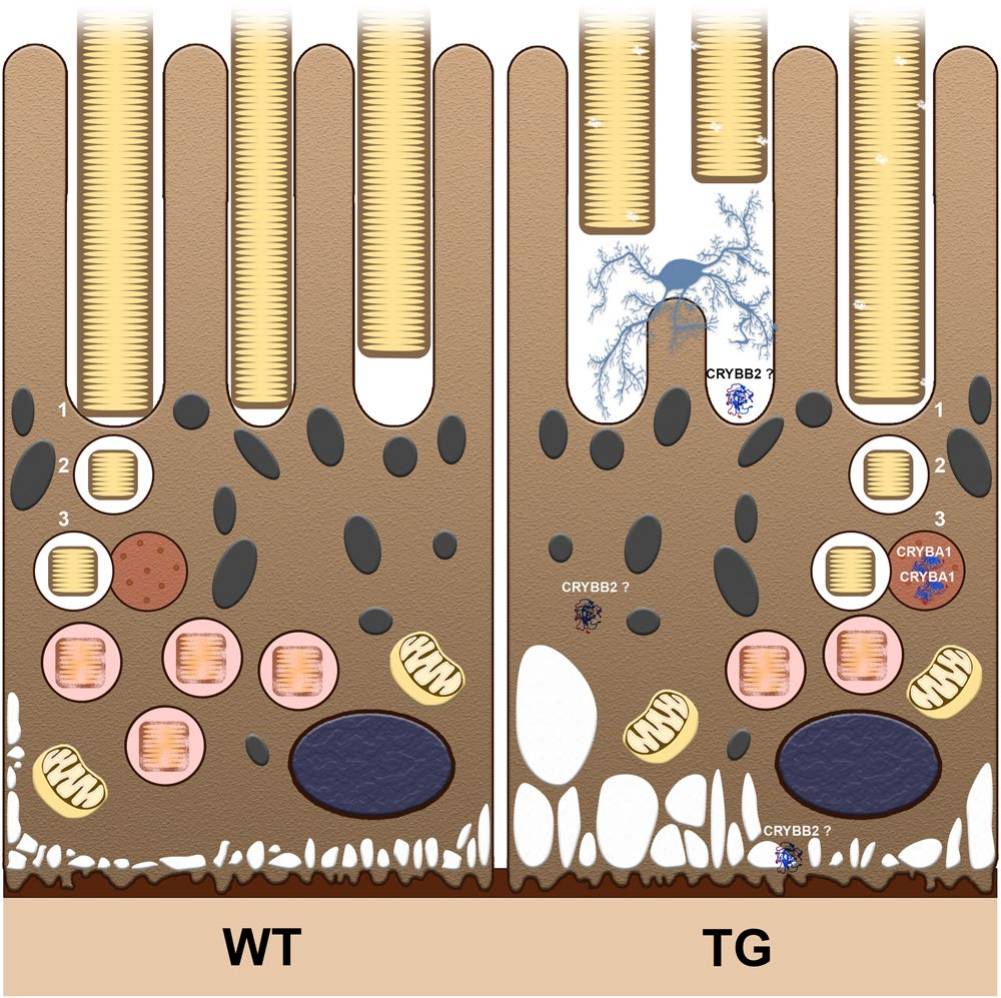
Summary of events occurring prior to photoreceptor death. Daily renewal of POS relies on phagocytosis of shed POS (in yellow) by underlying RPE cells (cytoplasm, brown; melanosomes, grey; nuclei, dark blue). In healthy cells (on the left), POS are recognized by microvilli (1) and phagosomes containing shed POS are internalized (2). Finally (3), phagosomes fuse with lysosomes (red vesicle), forming mature acidified phagolysosomes (red vesicles containing POS). In TG mice (on the right), photoreceptors expressing the mutant human ELOVL4 protein have outer segment ultrastructural abnormalities. Presentation of these segments to RPE cells leads to impaired phagolysosome maturation. Less acidified phagolysosomes are detected during the burst of phagocytic activity (2 hours after light onset). Lysosomal protein CRYBA1/A3 levels remain abundant (3 and 5 hours after light onset). Vacuoles (in white) form at the basal RPE. RPE cells produce CRYBB2, a protein present in AMD drusen. Phagocytic cells (microglia/macrophages, pale blue) are recruited to the subretinal space.

We describe early pathological events in a transgenic mouse model of STGD3 maculopathy, involving two phagocytic cell types, RPE and microglia/macrophages, prior to the death of photoreceptors. At the circadian peak of outer segment uptake, acidified RPE phagolysosomes are 50% less abundant in TG animals with photoreceptors expressing the mutant protein. This finding reveals that impairments are occurring in a step prior to degradation. The delays in degradation of fluorescent signal observed *in vitro*, when fluorescently labelled OS (isolated from TG compared with WT animals) are presented to human RPE cells may result from dysfunction in upstream events. *In vivo* evidence for processing delays at one month of age include: 1) increased expression of crystallin protein family member CRYBA1/A3, a regulator of lysosomal function^15^; 2) upregulation of drusen component CRYBB2^26^; and 3) invasion of microglia/macrophage in the central retina, where photoreceptor loss begins^4,29^, despite still undetectable accumulation of debris^8^; Fig. 6 summarizes these events.

Mouse models of RPE phagolysosomal maturation defects, such as induced in the absence of caveolin-1, share a phenotype of reduced lysosomal acidification and delayed POS protein clearance^31^. In *Abca4*−/− mice, lysosomal pH is abnormally elevated as early as four months, in parallel with age-dependent accumulation of A2E, the main constituent of lipofuscin^32^. Since we previously demonstrated normal A2E levels at one month of age in ELOVL4 transgenic mice (with increases of 10% at 3 months)^18^, this toxin is unlikely to affect lysosomal pH at this early stage^33^.

The long term impact on RPE homeostasis of daily occurring phagolysosomal maturation delays may provide an explanation for RPE vacuolization in TG mice observed already at one month^8^. Vacuolization has been reported in mouse models exhibiting dry-AMD pathological hallmarks including mutant *Elovl4* knock-in^34^. Vacuoles are also evident in models of lysosomal clearance defects, such as *Cryba1* cKO mice^35^.

Crystallins (alpha-, beta- and gamma-) have been associated with innate stress responses in neurodegenerative disorders^36^. Persistently elevated CRYBA1/A3 supports that compensatory homeostatic mechanisms are activated in RPE cells experiencing processing delays in the presence of photoreceptors expressing mutant ELOVL4 protein. CRYBB2, along with CRYBA4 and CRYGS accumulate in AMD drusen^26^. Upregulation of *Crybb2* and other crystallin genes was also reported following induction of RPE damage in D-galactose fed mice^24^. Whether CRYBB2 also accumulates in subretinal and/or basal laminar debris remains to be elucidated.

The IBA-1+ cells present in the subretinal space can be either microglia or macrophages or both^30^. Defects in RPE phagocytosis, and not cell death, have been attributed to the recruitment of microglia/macrophage cells in the subretinal space. As the primary resident immune cells in the retina, migrating microglial/macrophages initially participate in debris clearance^37^ and adaptive para-inflammation^38^. With the persistent build-up of undigested material, microglia/macrophages have been shown to impair the structure and physiology of RPE cells^39^ and to induce the expression by RPE cells of pro-inflammatory cytokines that aggravate microglia/macrophage accumulation, leading to deleterious inflammation and cell death^40^.

Disturbances in mitochondrial physiology are involved in a variety of neurodegenerative diseases^41^. Ultrastructural analysis of human AMD donor eyes revealed a reduction in the number of RPE mitochondria, associated with loss of cristae and membrane density^42^. We previously reported abnormal morphology in RPE mitochondria (swollen intercristal spaces) but only after the onset of photoreceptor death^8^. A recent study showed that photoreceptor outer segment phagocytic function provides the metabolic substrates needed for mitochondrial fatty acid β-oxidation and ketogenesis in the RPE^43^. In *Mreg*−/− and *Abca4*−/− mouse models, defective phagosome maturation and degradation lead to delayed ketogenesis^44^.

Our findings reveal that phagolysosomal maturation defects provide the earliest known link between photoreceptoral expression of human mutant ELOVL4 and the onset of photoreceptor cell death, and as such present a potential novel preventative target for STGD3 and other pathologies such as AMD that might share similar triggering events.

## Methods

### Animals

The animals used in this study were heterozygous transgenic ELOVL4 (TG1–2; TG) and wild-type (WT) littermate mice bred from a colony maintained at the University of Alberta, originally derived from the TG2 line generated by Karan *et al*.^29^, but with transgene expression levels comparable to the TG1 line^18^. All mice were culled at one month of age, with the exception of photoreceptor outer segment isolation, which gave optimal yield when using 2 month-old mice (due to larger eye size). Timing of light onset and subsequent tissue collection was strictly controlled to examine specific time points in relation to the peak of phagocytosis (2 hours into daylight cycle)^45^. They were housed under a 14:10 light-dark cycle, at 21 ± 2 °C, relative humidity 40% with ad libitum access to Laboratory Rodent Diet (5021; LabDiet®, Nutrition Intl., Richmond, IN) and water. All procedures were approved by the University of Alberta Animal Care and Use Committee (ACUC, license AUP00000328) and were conducted in accordance with the ARVO (Association for Research in Vision and Ophthalmology) Statement for the Use of Animals in Ophthalmic and Vision Research.

### Cell culture

ARPE-19 human stable cells (ATCC^®^ CRL2302^TM^) were cultured to optimize native phenotypes^46^. Post-confluent cultures in T75 flasks (353136, Falcon) were maintained in DMEM-F12 (11330–032, Gibco) supplemented with 100 U/mL penicillin, 100 μg/mL streptomycin (15140-122 Gibco) and 10% fetal bovine serum (Canadian origin, F1051, Sigma-Aldrich) at 37 °C in a humidified atmosphere of 5% CO_2_. Cells were split 1:2 every 4 weeks to allow epithelial phenotype maintenance^47^. Under these conditions, ARPE19 cells recapitulated all steps of OS phagocytosis, but with slower kinetics than in primary RPE cultures, as previously reported by Mazzoni *et al*.^48^. We followed their recommendations of 2–5 h as being optimal in cell lines for POS challenge (as opposed to 30 min to 2 h for unpassaged primary rat or mouse cell lines).

### Isolation of photoreceptor outer segments

Mouse photoreceptor outer segments (POS) were prepared using OptiPrep^TM^ density gradient medium (D1556, Sigma-Aldrich) as previously described^49^. *In vitro* quantification of POS phagocytosis by human RPE cells was performed as previously described^19^. In brief, eyes were dissected under dim red light and retinas collected in 120 µL ice-cold 8% OptiPrep^TM^ diluted in Ringer’s buffer, then vortexed at maximum speed for one minute, centrifuged at 200 × *g* for one minute and the supernatant was collected. This procedure was repeated five times to collect 700 µL of crude extract which was then loaded on a 10% and 18% OptiPrep^TM^ step gradient in Ultra-Clear^TM^ centrifuge tubes (347356, Beckman Coulter). Following centrifugation (30 minutes at 26,500 × *g*), the orange band containing POS on top of the 18% OptiPrep^TM^ was collected, diluted with four volumes of Ringer’s buffer and centrifuged at 500 × *g* for 3 minutes. The supernatant was transferred into a clean tube, centrifuged (26,500 × *g* for 30 minutes), the pellet collected and resuspended in 8% OptiPrep^TM^ to a final concentration of 1 × 10^7^ POS/mL and stored at −80 °C. Just before use, POS were labelled with 1% (v/v) Alexa Fluor 488 carboxylic acid succinimidyl ester (A20000, Molecular Probes, 15 mM) for one hour on ice, rinsed three times with PBS and finally resuspended in DMEM-F12 medium (11330-032, ThermoFisher Scientific) supplemented with 1% FBS (F1051, Sigma-Aldrich). POS concentration was verified by direct counting on a hemocytometer.

### Phagocytosis assay

Post-confluent stationary human RPE cells were seeded at a confluence of 20,000 cells per well in Nunc® MicroWell 96 well optical bottom plates (P8991, Sigma-Aldrich) and cultured for 2 weeks in DMEM-F12 medium (11330-032, ThermoFisher Scientific) supplemented with 1% FBS (F1051, Sigma-Aldrich). To quantify the binding and internalization of POS, cells were fed with Alexa-Fluor 488 labelled POS (10 POS per cell) and incubated for 30, 60, 90 and 120 minutes at 37 °C. Cells were then rinsed twice with ice-cold Dulbecco’s phosphate-buffered saline, no calcium, no magnesium (DPBS-CM, 14190-144, ThermoFisher Scientific). A subset of the wells were incubated with ice-cold trypan blue 0.4% for 10 minutes to quench the fluorescence derived from externally bound particles, and rinsed twice with ice-cold DPBS-CM. POS processing by human RPE cells was studied using pulse-chase assays. Following 2 hours incubation with Alexa-Fluor 488 labelled POS, cells were rinsed twice with growth medium to remove the unbound POS and the cells were then return to 37 °C for chase periods of 4, 8, 15, or 24 hours. Human RPE cells were fixed 10 minutes with ice-cold methanol and counterstained with ethidium bromide. Fluorescence signals were quantified in duplicate wells using a Typhoon Trio scanner (GE Healthcare). POS fluorescence was normalized using ethidium bromide fluorescence^50^. Mann-Whitney U-test (one-tailed) was used with significance set to P ≤ 0.05; n = 3–5 independent experiments. For each individual experiment, POS were isolated from 26 pooled retinas per group.

### Live imaging of LysoTracker-labelled phagolysosomes

Phagolysosomes were stained and imaged as previously described^19^. Freshly dissected posterior eyecups were incubated at 37 °C for 15 minutes in DMEM (11995-065, Gibco) with 0.4 µM LysoTracker^TM^ Red DND-99 (L7528, Molecular Probes) to label acidified phagolysosomes and 5 µM 4′,6-Diamidine-2′-phenylindole dihydrochloride (DAPI) to stain nuclei. Confocal images (z-stacks, 0.3 µm steps, 30 µm in total) were captured in the central part of the RPE, using an Olympus IX-81 inverted microscope equipped with a Yokagawa CSU X1 spinning disk confocal scan-head, a Hamamatsu EMCCD camera (C9100-13). The volume occupied by phagolysosomes inside individual RPE cells was calculated using Volocity™ software (version 6.3, PerkinElmer). RPE cells were imaged from 8 independent experiments (4 WT and 4 TG animals) and a total of 13 microscope fields per group (3–4 images per animal), representing 297 and 331 RPE cells from WT and TG animals respectively, for final analysis. Mann-Whitney U-test (one-tailed) was used; significance was set to P ≤ 0.05.

### Nuclear and cytoplasmic TFEB localization by western blotting

Nuclear and cytoplasmic proteins were isolated from freshly dissected posterior eyecups (RPE/choroid) using the NE-PER Nuclear and Cytoplasmic Extraction Reagent kit (#78833, ThermoFisher Scientific) according to the manufacturer instructions. Animals were culled 2 hours after light onset and homogenized tissues from 3–4 animals were pooled. Nuclear and cytoplasmic extracts were kept at −80 °C until use. Protein levels were determined (Pierce BCA protein assay kit, PI-23227, ThermoFisher Scientific). After addition of 4 × Laemmli sample buffer (161-0747, BioRad) with 2-mercaptoethanol (2.5% final conc.), samples were boiled for 5 minutes, 2 µg total protein (nuclear extract) or 5 µg total protein (cytoplasmic extract) were loaded per lane on 10% SDS-PAGE gels, run at 200 V constant voltage for 60 minutes, and then transferred to PVDF membranes. After blocking for 1 hour in 5% skim milk powder in TBS-T (20 mM Tris, 137 mM NaCl, pH 7.6 with 0.1% Tween-20), membranes were incubated overnight with rabbit polyclonal anti-TFEB (1:5000, A303-673A, Bethyl Laboratories) diluted in block solution. The following day membranes were washed 3 × 10 minutes in TBS-T and incubated for 1 hour with anti-rabbit IgG HRP-conjugated antibody (1:5000 in block; NA934, GE Healthcare) as before. Then membranes were washed 2 × 10 minutes in TBS-T, 2 × 5 minutes in TBS (no Tween-20), and visualized with Clarity™ Western ECL substrate (170-5060 BioRad) on a ChemiDoc™ Touch Imaging System (BioRad). ImageLab version 5.2.1 software (BioRad) was used to determine volume (intensity) of bands (samples loaded in duplicate, 2 independent experiments). Isolation of cytoplasmic and nuclear proteins specifically was verified using PCNA (1:500, rabbit polyclonal, ab2426, Abcam) and TUBA (1:500, mouse monoclonal, sc-8035, Santa Cruz), respectively. Total protein loading and transfer was verified by staining the blot with GelCode® Blue stain reagent (24590, ThermoFisher Scientific). TFEB signal was measured in duplicate wells for each sample and the ratio of cytoplasmic over nuclear TFEB (for 5 µg of total protein) was calculated in two independent experiments.

### RPE flatmounts and immunohistochemistry

After light 4% PFA fixation and sucrose cryoprotection, eyecups (cornea and lens removed) were embedded in OCT (Tissue-Tek, Sakura® Finetek) and stored at −80 °C until flatmount preparation. After eyecups were washed in PBS and flattened with four radial cuts, whole retinas were gently removed. RPE flatmounts were blocked for 1 hour in PBS with 0.3% Triton X-100, 0.1% Tween-20, and 5% bovine serum albumin and then reacted overnight at 4 °C with primary antibodies diluted in same: rabbit polyclonal anti-TFEB (1:500, A303-673A, Bethyl Laboratories); or rabbit polyclonal anti-IBA-1 (1:400, 019-19741, Wako). F-actin was detected by incubation with Alexa Fluor 488 Phalloidin (1:40, A12379, Molecular Probes) for 30 minutes. After extensive PBS washes, RPE flatmounts were incubated for 2 hours with 10 µg/mL Hoechst (Bisbenzimide, H33258, Sigma-Aldrich) and 1:1000 species appropriate Alexa Fluor labelled secondary antibodies (Molecular Probes). RPE flatmounts were washed as before, then cover-slipped and mounted with Vectashield mounting media (H-1000, Vector Laboratories). TFEB immunoreactivity was evaluated on the central part of the RPE flatmount (mice were culled 3 hours after light onset). Total numbers of IBA-1 positive cells were counted. Mann-Whitney U-test was used; significance was set to P ≤ 0.05, with n = 4 animals per group.

### cDNA synthesis and quantitative RT-PCR

Total RNA was isolated immediately following RPE and retina dissection. RNA was reverse transcribed and cDNA quantified using real-time PCR. All details regarding nucleic acid extraction, reverse transcription, qPCR assay settings and validation in adherence with MIQE guidelines are respectively described in Supplementary Tables 1 and 2. Expression of CLEAR genes (*Tfeb*, *Map1lc3a*, *Atp6v0a1* and *Ctsd*), chemokine genes (*Ccl2*, *Ccl5*) and crystallin family genes (*Cryaa*, *Cryab*, *Cryba1*, *Cryba4*, *Crybb2*, *Cryga* and *Crygs*) was normalized to the reference gene *Hprt*. Student’s t-test and Mann-Whitney U-test were used appropriately. Data are presented as mean ± SEM. Significance was set to P ≤ 0.05; n = 5–10 animals per group.

### RPE cell protein preparation and western blotting

For LC3B-I/LC3B-II, cathepsin D and beta-crystallin A1/A3 western blots, animals were culled 3 hours or 5 hours after light onset (n = 4–5 animals). Proteins were prepared from isolated RPE as previously described (confirmed RPE markers with an absence of choroid markers)^50^.

Briefly, after removing neural retina, four slits were made in the posterior eyecup and the flattened tissue was immersed in 100 µL RIPA lysis buffer (R0278, Sigma-Aldrich) with HALT™ Protease inhibitor cocktail (87786, ThermoFisher Scientific) on ice. Each tube was tapped 20 times and returned to ice, and this was repeated 5 times in total (100 taps each to release RPE). After incubation on ice for a maximum of 20 minutes (to prevent choroid contamination), choroid/sclera was discarded, and RPE from individual animals was pooled (200 µL per tube). Samples were then sonicated 2 × 10 seconds on ice. After centrifugation, 14,000 rpm for 15 minutes at 4 °C, supernatants were collected and stored at −80 °C until use. Protein concentrations were measured and 10 µg of total protein per lane were analysed on 12% SDS-PAGE gels and PVDF membranes as described in the previous section. Membranes were incubated overnight with LC3B (1:1000, rabbit polyclonal, 2775, Cell Signaling Technology), cathepsin D (1:500, goat polyclonal, sc-6486, Santa Cruz) or beta-crystallin A1/A3 (1:5000, rabbit polyclonal, PA5-28954, ThermoFisher Scientific), diluted in block solution. Membranes were washed, incubated 1 hour with anti-rabbit or anti-goat IgG HRP-conjugated antibody (1:5000 in block solution; NA934, GE Healthcare; sc-2354 Santa Cruz, respectively), washed again and imaged as described before (samples loaded in duplicate, 2 independent experiments). Protein levels were normalized to α-tubulin (TUBA, 1:500, mouse monoclonal, sc-8035, Santa Cruz) loading control.

### Mass spectrometry

Posterior eyecups (RPE/choroid) collected from TG and WT (n = 6–8 each per experiment, run in duplicate) were pooled and homogenized in sample lysis buffer (20 mM Tris HCl, pH 7.5; 150 mM NaCl; 1 mM EGTA; 1% Triton X-100, HALT™ Protease inhibitor cocktail, 87786, ThermoFisher Scientific). After 30 minutes at 4 °C, samples were passed repeatedly through a 26-gauge needle, centrifuged 5 minutes at 4 °C and supernatants were frozen at −80 °C until use. Protein levels were determined (Pierce BCA protein assay kit, PI-23227, ThermoFisher Scientific). After addition of 2% (vol/vol) 2-mercaptoethanol and 1% (vol/vol) saturated bromophenol blue, samples were boiled for 5 minutes, 20 µg total protein was loaded per lane on 12% SDS-PAGE gels, and gels were run at 200 V constant voltage for 45 minutes. Gels were western blotted for beta-crystallin A1/A3 as described above. Duplicate gels were washed in ultrapure water (3 × 5 minutes), stained for one hour with GelCode® Blue stain reagent (24590, ThermoFisher Scientific), and rinsed with ultrapure water. Mass spectrometry was performed by the Alberta Proteomics and Mass Spectrometry Facility (Department of Biochemistry, Faculty of Medicine & Dentistry, University of Alberta).

Excised gel lanes were de-stained twice in 100 mM ammonium bicarbonate, acetonitrile (50:50). The samples were then reduced (10 mM βME in 100 mM bicarbonate) and alkylated (55 mM iodoacetamide in 100 mM bicarbonate). After dehydration, in-gel trypsin digestion was allowed to proceed overnight at room temperature. Tryptic peptides were first extracted from the gel using 97% water, 2% acetonitrile, 1% formic acid followed by a second extraction using 50% of the first extraction buffer and 50% acetonitrile. Fractions containing tryptic peptides were resolved and ionized by using nanoflow HPLC (Easy-nLC II, ThermoFisher Scientific) coupled to an LTQ XL-Orbitrap hybrid mass spectrometer (ThermoFisher Scientific). Nanoflow chromatography and electrospray ionization were accomplished by using a PicoFrit fused silica capillary column (ProteoPepII, C18) with 100 μm inner diameter (300 Å, 5 μm, New Objective). Peptide mixtures were injected onto the column at a flow rate of 3,000 nL/minutes and resolved over 60 minutes at 500 nL/minutes using linear gradients from 0 to 45% v/v aqueous ACN in 0.2% v/v formic acid. The mass spectrometer was operated in data-dependent acquisition mode, recording high-accuracy and high-resolution survey Orbitrap spectra using external mass calibration, with a resolution of 30,000 and m/z range of 400–2000. The fourteen most intense multiply charged ions were sequentially fragmented by using collision induced dissociation and spectra of their fragments were recorded in the linear ion trap; after two fragmentations all precursors selected for dissociation were dynamically excluded for 60 seconds.

Raw data were processed using the proteomic platform MaxQuant version 1.5.8.2 (http://maxquant.org) with the computational workflow designed for quantitative label-free proteomics^51^ and data were analysed with Perseus version 1.5.8.5. Raw MaxQuant files were searched against Uniprot *Mus musculus* reference proteome database (UP000000589, strain C57BL/6J). Data were filtered to keep only proteins detected in all samples. Label-free quantification (LFQ) methodology was used to compare the levels of identified proteins between TG and WT. To estimate the abundance of identified proteins, LFQ intensities were transformed by Log_2_ to fit a normal distribution then subjected to hierarchical clustering and presented as multiscatter plots.

### High-resolution respirometry

To provide a sensitive measure of oxidative stress and assess the bioenergetics status of RPE cells (essential for phagocytosis), high-resolution respirometry (Oxygraph 2k, OROBOROS Instruments, Innsbruck, Austria) was performed on freshly isolated TG and WT eyecups (2 littermates/4 eyecups pooled per measurement), n = 8 measurements for each genotype, with slight modification from the method previously described^29^. In brief, after measurement of wet weight, tissues were immediately transferred into 1 mL of ice-cold MiR05 (0.5 mM EGTA, 3 mM MgCl_2_∙6H_2_O, 60 mM K-lactobionate, 20 mM taurine, 10 mM KH_2_PO_4_, 20 mM HEPES, 110 mM sucrose and 1 g/L BSA essentially fatty acid free, pH 7.1^55^ and homogenized on ice with a Potter-Elvehjem attached to an overhead stirrer (Wheaton Instruments). After ten passes (intensity level two), 600 µL of homogenate was immediately placed in an oxygraph chamber containing 1.4 mL of MiR05. Remaining homogenate was frozen at −80 °C for measurement of citrate synthase (CS) activity and total protein using a BCA protein assay (PI-23227, ThermoFisher Scientific).

The protocol used for evaluating mitochondrial function included sequential addition of the following substrates and inhibitors: pyruvate (5 mM), malate (5 mM), ADP (2.5 mM), cytochrome *c* (cyt. *c*; 10 mM), succinate (10 mM), rotenone (1 μM), antimycin A (5 μM), ascorbate (2 mM), tetramethylphenylenediamine (TMPD; 0.5 mM), and azide (100 mM). Residual oxygen consumption (ROX; non-mitochondrial oxygen consumption), measured after inhibition of Complexes I and III with rotenone and antimycin A represented a small fraction (median of 5%) of maximal NS-OXPHOS capacity and was subtracted. For Complex IV respiration (CIV, cytochrome *c* oxidase), chemical background measured in the presence of sodium azide was also subtracted. Respiration was expressed in flux per mass of tissue and as Flux Control Ratios, FCR, normalized for maximal NS-OXPHOS capacity.

Datlab software (OROBOROS Instruments, Innsbruck, Austria) was used for data acquisition and analysis. Using SigmaStat4 (Aspire Software International) software, each variable was tested for normality and homogeneity of variance for ANOVA with Kolmogorov-Smirnov (Lilliefors’ correction) and Spearman tests, respectively. Student’s t-test and Mann-Whitney U-test were used appropriately. Data are presented as mean ± SEM. Significance was set to P ≤ 0.05; n = 8 independent experiments.

## Electronic supplementary material

Supplementary Information

## References

[1] Agbaga MP (2008). Role of Stargardt-3 macular dystrophy protein (ELOVL4) in the biosynthesis of very long chain fatty acids. Proc. Natl. Acad. Sci. USA.

[2] Grayson C, Molday RS (2005). Dominant negative mechanism underlies autosomal dominant Stargardt-like macular dystrophy linked to mutations in ELOVL4. J. Biol. Chem..

[3] Barabas P (2013). Role of ELOVL4 and very long-chain polyunsaturated fatty acids in mouse models of Stargardt type 3 retinal degeneration. Proc. Natl. Acad. Sci. USA.

[4] Kuny S, Gaillard F, Sauvé Y (2012). Differential gene expression in eyecup and retina of a mouse model of Stargardt-like macular dystrophy (STGD3). Invest. Ophthalmol. Vis. Sci..

[5] Logan S (2013). Deciphering mutant ELOVL4 activity in autosomal-dominant Stargardt macular dystrophy. Proc. Natl. Acad. Sci. USA.

[6] Bennett LD (2014). Effect of reduced retinal VLC-PUFA on rod and cone photoreceptors. Invest. Ophthalmol. Vis. Sci..

[7] Agbaga MP (2014). Mutant ELOVL4 that causes autosomal dominant stargardt-3 macular dystrophy is misrouted to rod OS disks. Invest. Ophthalmol. Vis. Sci..

[8] Kuny S, Cho WJ, Dimopoulos IS, Sauvé Y (2015). Early Onset Ultrastructural and Functional Defects in RPE and Photoreceptors of a Stargardt-Like Macular Dystrophy (STGD3) Transgenic Mouse Model. Invest. Ophthalmol. Vis. Sci..

[9] Sparrow JR (2012). The bisretinoids of retinal pigment epithelium. Prog. Retin. Eye Res..

[10] Krock BL, Bilotta J, Perkins BD (2007). Non cell-autonomous photoreceptor degeneration in a zebrafish model of choroideremia. Proc. Natl. Acad. Sci. USA.

[11] Young RW, Bok D (1969). Participation of the retinal pigment epithelium in the rod outer segment renewal process. J. Cell Biol..

[12] Duncan JL (2003). An RCS-like retinal dystrophy phenotype in mer knockout mice. Invest. Ophthalmol. Vis. Sci..

[13] Picard E (2010). CD36 plays an important role in the clearance of oxLDL and associated age-dependent sub-retinal deposits. Aging (Albany N.Y.).

[14] Zhang D (2005). Correlation between inactive cathepsin D expression and retinal changes in mcd2/mcd2 transgenic mice. Invest. Ophthalmol. Vis. Sci..

[15] Zigler JS (2011). Mutation in the βA3/A1-crystallin gene impairs phagosome degradation in the retinal pigmented epithelium of the rat. J. Cell Sci..

[16] Kinnunen K, Petrovski G, Moe MC, Berta A, Kaarniranta K (2012). Molecular mechanisms of retinal pigment epithelium damage and development of age-related macular degeneration. Acta Ophthalmol..

[17] Wong WL (2014). Global prevalence of age-related macular degeneration and disease burden projection for 2020 and 2040: a systematic review and meta-analysis. Lancet Glob. Health.

[18] Kuny S (2010). Inner retina remodeling in a mouse model of Stargardt-like macular dystrophy (STGD3). Invest. Ophthalmol. Vis. Sci..

[19] Mao Y, Finnemann SC (2016). Live imaging of LysoTracker-labelled phagolysosomes tracks diurnal phagocytosis of photoreceptor outer segment fragments in rat RPE tissue *ex vivo*. Adv. Exp. Med. Biol..

[20] Xu J, Toops K, Lakkaraju A (2013). Photoreceptor outer segment phagocytosis couples lysosome biogenesis to autophagy in the retinal pigment epithelium. Invest. Ophthalmol. Vis. Sci..

[21] Sardiello M (2009). A gene network regulating lysosomal biogenesis and function. Science.

[22] Settembre C (2012). A lysosome-to-nucleus signalling mechanism senses and regulates the lysosome via mTOR and TFEB. EMBO J..

[23] Settembre C (2011). TFEB links autophagy to lysosomal biogenesis. Science.

[24] Tian J (2005). Advanced glycation endproduct-induced aging of the retinal pigment epithelium and choroid: a comprehensive transcriptional response. Proc. Natl. Acad. Sci. USA.

[25] Yaung J (2007). alpha-Crystallin distribution in retinal pigment epithelium and effect of gene knockouts on sensitivity to oxidative stress. Mol. Vis..

[26] Crabb JW (2002). Drusen proteome analysis: an approach to the etiology of age-related macular degeneration. Proc. Natl. Acad. Sci. USA.

[27] Gnaiger E, Lassnig B, Kuznetsov A, Rieger G, Margreiter R (1998). Mitochondrial oxygen affinity, respiratory flux control and excess capacity of cytochrome *c* oxidase. J. Exp. Biol..

[28] Han WH (2017). Modifications in Retinal Mitochondrial Respiration Precede Type 2 Diabetes and Protracted Microvascular Retinopathy. Invest. Ophthalmol. Vis. Sci..

[29] Karan G (2005). Lipofuscin accumulation, abnormal electrophysiology, and photoreceptor degeneration in mutant ELOVL4 transgenic mice: a model for macular degeneration. Proc. Natl. Acad. Sci. USA.

[30] Langmann T (2007). Microglia activation in retinal degeneration. J. Leukoc. Biol..

[31] Sethna S (2016). Regulation of Phagolysosomal Digestion by Caveolin-1 of the Retinal Pigment Epithelium Is Essential for Vision. J. Biol. Chem..

[32] Mata NL (2001). Delayed dark-adaptation and lipofuscin accumulation in abcr +/− mice: implications for involvement of ABCR in age-related macular degeneration. Invest. Ophthalmol. Vis. Sci..

[33] Bergmann M, Schütt F, Holz FG, Kopitz J (2004). Inhibition of the ATP-driven proton pump in RPE lysosomes by the major lipofuscin fluorophore A2-E may contribute to the pathogenesis of age-related macular degeneration. FASEB J..

[34] Vasireddy V (2009). Elovl4 5-bp deletion knock-in mouse model for Stargardt-like macular degeneration demonstrates accumulation of ELOVL4 and lipofuscin. Exp. Eye Res..

[35] Shang P (2017). The amino acid transporter SLC36A4 regulates the amino acid pool in retinal pigmented epithelial cells and mediates the mechanistic target of rapamycin, complex 1 signaling. Aging Cell.

[36] Fort PE, Lampi KJ (2011). New focus on alpha-crystallins in retinal neurodegenerative diseases. Exp. Eye Res..

[37] Joly S (2009). Cooperative phagocytes: resident microglia and bone marrow immigrants remove dead photoreceptors in retinal lesions. Am. J. Pathol..

[38] Xu H, Chen M, Forrester JV (2009). Para-inflammation in the aging retina. Prog. Retin. Eye Res..

[39] Ma W, Zhao L, Fontainhas AM, Fariss RN, Wong WT (2009). Microglia in the mouse retina alter the structure and function of retinal pigmented epithelial cells: a potential cellular interaction relevant to AMD. PLoS One..

[40] Kohno H (2013). Photoreceptor proteins initiate microglial activation via Toll-like receptor 4 in retinal degeneration mediated by all-trans-retinal. J. Biol. Chem..

[41] Golpich M (2017). Mitochondrial Dysfunction and Biogenesis in Neurodegenerative diseases: Pathogenesis and Treatment. CNS Neurosci. Ther..

[42] Feher J (2006). Mitochondrial alterations of retinal pigment epithelium in age-related macular degeneration. Neurobiol. Aging.

[43] Reyes-Reveles J (2017). Phagocytosis-dependent ketogenesis in retinal pigment epithelium. J. Biol. Chem..

[44] Di Pierdomenico J (2017). Early Events in Retinal Degeneration Caused by Rhodopsin Mutation or Pigment Epithelium Malfunction: Differences and Similarities. Front. Neuroanat..

[45] LaVail MM, Rod OS (1976). disk shedding in rat retina: relationship to cyclic lighting. Science.

[46] Samuel W (2017). Appropriately differentiated ARPE-19 cells regain phenotype and gene expression profiles similar to those of native RPE cells. Mol. Vis..

[47] Mao Y, Finnemann SC (2013). Analysis of photoreceptor outer segment phagocytosis by RPE cells in culture. Methods Mol. Biol..

[48] Mazzoni F, Safa H, Finnemann SC (2014). Understanding photoreceptor outer segment phagocytosis: use and utility of RPE cells in culture. Exp. Eye Res..

[49] Tsang SH (1998). Role for the target enzyme in deactivation of photoreceptor G protein *in vivo*. Science.

[50] Wei H, Xun Z, Granado H, Wu A, Handa JT (2016). An easy, rapid method to isolate RPE cell protein from the mouse eye. Exp. Eye Res..

[51] Tyanova S, Temu T, Cox J (2016). The MaxQuant computational platform for mass spectrometry-based shotgun proteomics. Nat.Protoc..

