# Supplementary material for: Photoreceptor-induced RPE phagolysosomal maturation defects in Stargardt-like Maculopathy (STGD3)
Source: Sci Rep. 2018 Apr 13;8:5944. doi: 10.1038/s41598-018-24357-4 (PMC5899129; doi:10.1038/s41598-018-24357-4)
Supplement: Supplementary file 1 — Supplementary Information [file 41598_2018_24357_MOESM1_ESM.pdf]

Supplementary Information

Photoreceptor-induced RPE phagolysosomal maturation defects in Stargardt-like Maculopathy (STGD3)

Camille Dejos[1], Sharee Kuny[1], Woo Hyun Han[1], Heather Capel[2], Hélène Lemieux[3], Yves Sauvé[1,2]*

[1]Department of Ophthalmology and Visual Sciences, 7-45 Medical Sciences Building, University of Alberta, Edmonton, AB, T6G 2H7, Canada

[2]Department of Physiology, 7-45 Medical Sciences Building, University of Alberta, Edmonton, AB, T6G 2H7, Canada

[3]Faculty Saint-Jean, 8406 Rue Marie-Anne Gaboury Northwest, University of Alberta, Edmonton, AB T6C 4G9, Canada

* Corresponding author: Yves Sauvé, Department of Physiology, 7-45 Medical Sciences Bldg; University of Alberta, Edmonton AB, Canada, T6G 2H7; Tel: 780-492-8609; Fax: 780-248-1995; E-mail: ysauve@ualberta.ca

Supplementary Figure 1. Full-length of the cropped blots presented in Figure 2 panel A of the manuscript. Immunoblots of TFEB, PCNA and TUBA using cytoplasmic proteins (5 µg per lane) and nuclear proteins (2 µg per lane) from RPE homogenates prepared 2 hours after light onset (7AM).

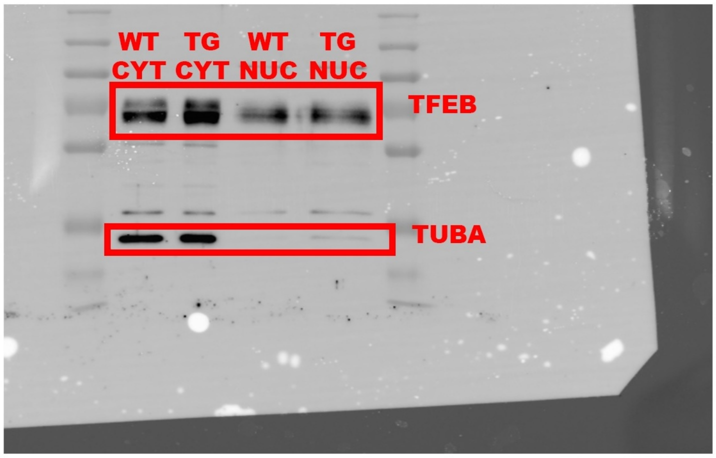

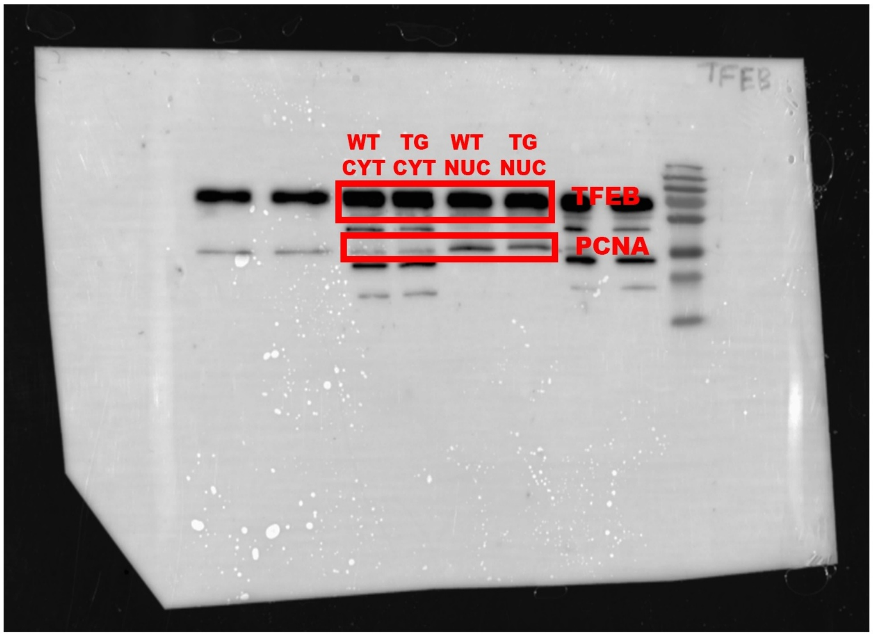

Supplementary Figure 2. Full-length of the cropped blots presented in Figure 2 panel D of the manuscript. Immunoblots of LC3B-I/LC3B-II, CTSD (i, immature and m, mature) and TUBA using RPE homogenates prepared 3 hours (8AM) and 5 hours (10AM) after light onset.

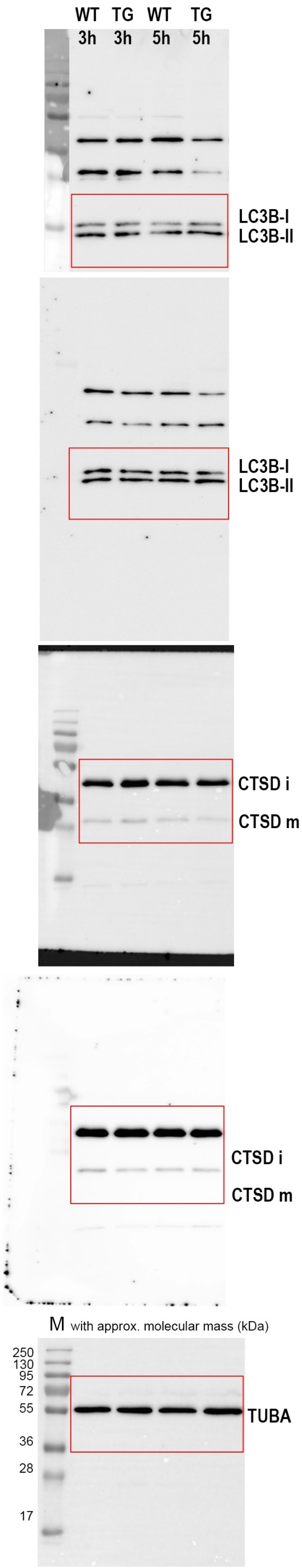

Supplementary Figure 3. Full-length of the cropped blots presented in Figure 3 panel D of the manuscript. Immunoblots of CRYBA1/3 and TUBA using RPE/choroid homogenates analysed by mass spectrometry (left, proteins from 3-4 mice per lane) and isolated RPE protein extracts (right, 1 animal per lane, n = 4 each group) with graph (far right) of CRYBA1/3 net intensity levels corrected for loading.

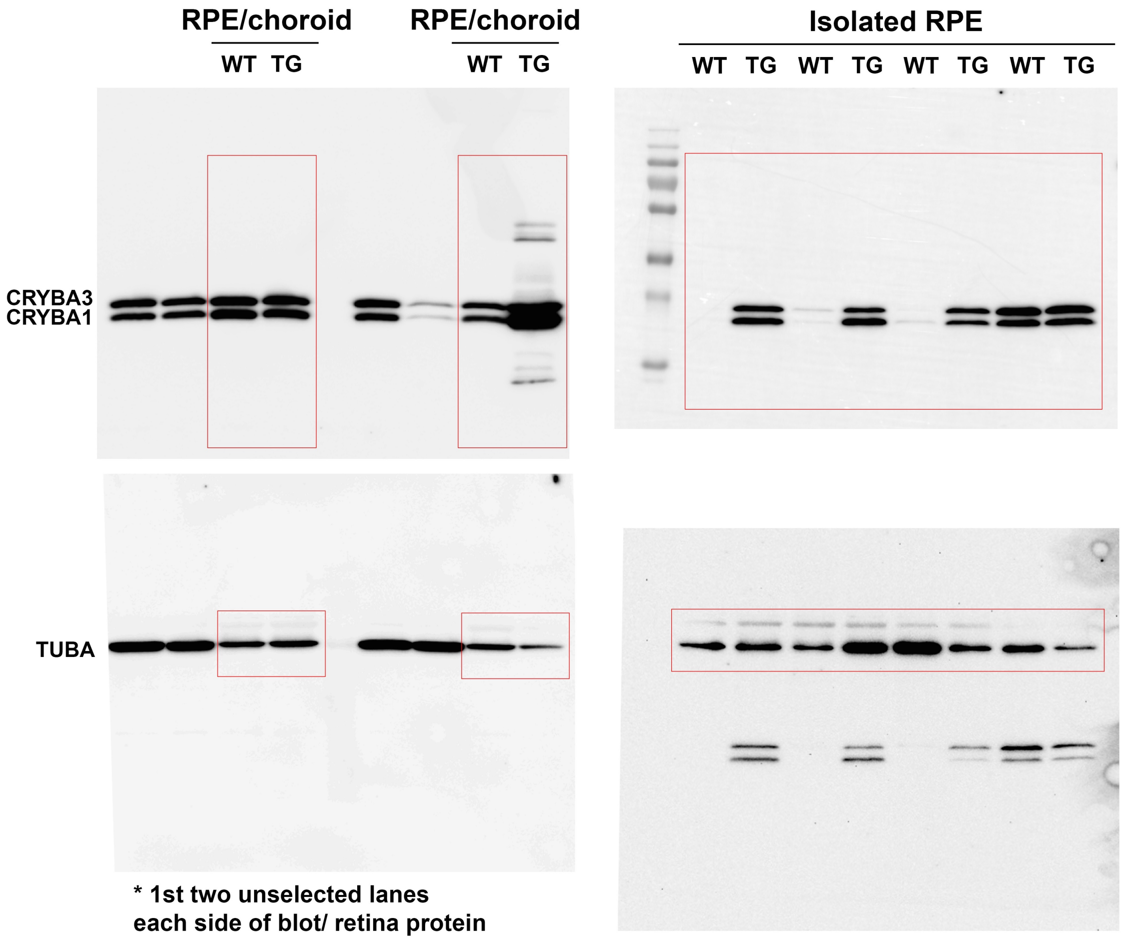

Supplementary Figure 4. Flatmounts of RPE from one month old WT and TG mice, stained using F-actin (green) and Hoechst (blue).

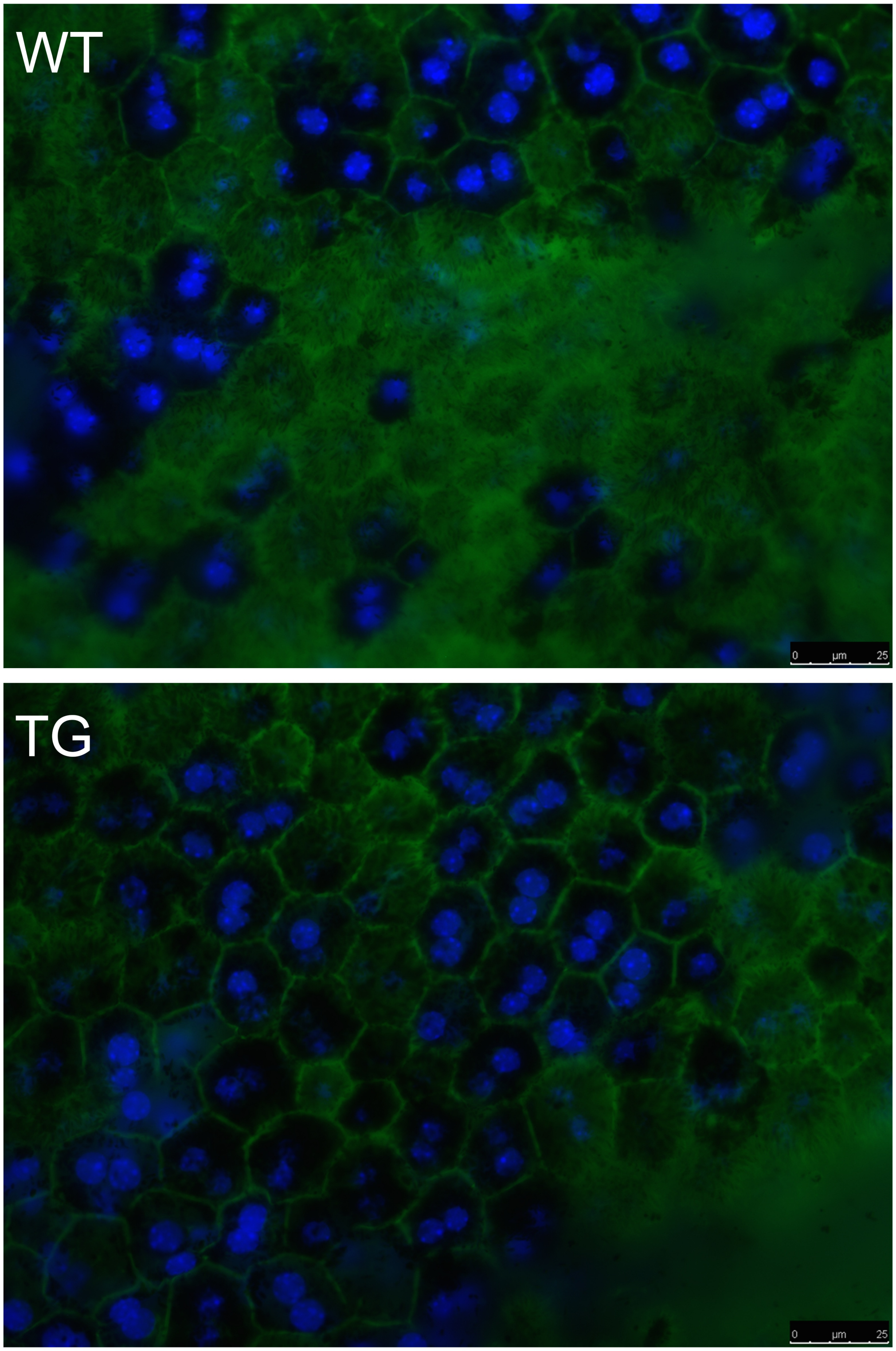

Supplementary Table 1. Description of primers and amplicons used in quantitative RT-PCR assay.

Gene Name	NCBI Reference Sequence	Splice variants targeted	Forward Primer (5' to 3')	F Primer location	Reverse primer (5' to 3')	R Primer location	Amplicon
Hprt	**NM_013556.2**		**TTG GAT ACA GGC CAG ACT TTG**	**Exon 6**	**TGG CAA CAT CAA CAG GAC TC**	**Exon 8**	**194 bp**
Tfeb	**NM_011549.3**	**1, 2, 3, 4**	**GAC GCA GGT TCA ACA TCA ATG**	**Exon 5**	**CTT GTT AGT CAT CTC CAG GCG**	**Exon 6**	**194 bp**
Map1lc3a	**NM_025735.3**	**1, 2**	**TCC TGG ATA AGA CCA AGT TTC TG**	**Exon 3**	**CGT CTT CAT CCT TCT CCT GTT C**	**Exon 4**	**183 bp**
Atp6v0a1	**NM_016920.3**	**1, 2, 3, 4, 5, 6, 7, 8, 9**	**AAC ACT AAC CAG GAA GCT CTG**	**Exon**	**GTT AAT CAC ACC AGC CAC AAA G**	**Exon**	**195 bp**
Ctsd	**NM_009983.2**		**TGA CAA GTC CAG CAC CTA TG**	**Exon 4**	**CTC CAC CTT GAT ACC TCT TGC**	**Exon 5**	**145 bp**
Ccl2	**NM_011333.3**		**TGA ATG TGA AGT TGA CCC GT**	**Exon 3**	**TTA AGG CAT CAC AGT CCG AG**	**Exon 3**	**129 bp**
Ccl5	**NM_013653.3**		**GAA GAT CTC TGC AGC TGC CC**	**Exon 1**	**CTA GCT CAT CTC CAA ATA GTT G**	**Exon 3**	**274 bp**
Cryaa	**NM_001278570.1**	**1, 2, 3**	**GGT CCC TGC ATC ACC AAA TA**	**Exon 1**	**GCA GCA GGT CGT ACT CAA A**	**Exon 1**	**136 bp**
Cryab	**XM_006509970.3**	**1, 2, 3, 4**	**TCT TCT CAA CAG CCA CTT CC**	**Exon 2**	**TCC TTC TCC AAA CGC ATC TC**	**Exon 3**	**109 bp**
Cryba1	**NM_009965**	**1**	**CAG ACC AAC CCT ATG CCA G**	**Exon 2**	**TCC AGG ATG AAC TGT TGC C**	**Exon 4**	**211 bp**
Cryba4	**XM_006534749.3**	**1, 2**	**GGC TCC TTC CAT GTT CAA TCT**	**Exon**	**CAC TCT CTG AAG TGC TTG TAG TC**	**Exon**	**118 bp**
Crybb2	**NM_007773**	**1, 2, 3**	**CCA TTC CCA CGA GCT CAG**	**Exon 4**	**TCG CCC TTT TCA AAC ACA AAC**	**Exon 5**	**147 bp**
Cryga	**NM_007774.3**		**CAG CAG TGG ATG GGT TTC A**	**Exon 2**	**CCC GGT AGT CAT CTC TCT CAT A**	**Exon 3**	**100 bp**
Crygs	**NM_009967.2**	**1**	**AGT CGC TGC AAC TCC ATT AG**	**Exon 2**	**CCA TCC AAC GCT GGT ATT CA**	**Exon 2**	**121 bp**

Supplementary Table 2. MIQE checklist**.**

Item to check	Importance	
**Experimental design**		
Definition of experimental and control groups	E	Experimental groups: TG mice culled 3 hours and 5 hours after light onset. Control groups: WT mice culled 3 hours and 5 hours after light onset.
Number within each group	E	*n* = 5 to 10
Assay carried out by the core or investigator's laboratory?	D	Investigator's laboratory
Acknowledgment of author's contribution	D	The experiments were designed by Dejos C. and executed by Dejos C. and Capel H.
**Samples**		
Description	E	Experimental groups: neural retinas or eyecups from TG mice culled 3 hours and 5 hours after light onset. Control groups: neural retinas or eyecups from WT mice culled 3 hours and 5 hours after light onset.
Volume/mass of sample processed	D	Mouse retina P30: 1.5 mg. Mouse eyecup P30: 3 mg
Microdissection or macrodissection	E	macrodissection
Processing procedure	E	Mice are euthanized with intraperitoneal injection of euthanyl before enucleation.
If frozen, how and how quickly?	E	Not frozen
If fixed, with what and how quickly?	E	Not applicable
Sample storage conditions and duration	E	Not stored. Freshly dissected tissues were immediately used for nucleic acid extraction.
**Nucleic acid extraction**		
Procedure and/or instrumentation	E	Immediately following dissection, total RNA was isolated from single posterior eyecups harvested in 200 µL of RNAprotect (76506, Qiagen) following a previously described method (Wang et al. Exp Eye Res 2012;102:1e9). Total RNA from RPE and retinas were isolated using an RNeasy micro kit (74004, Qiagen) and DNase I treatment was performed following manufacturer's recommendations. Purified total RNA was eluted in RNAse-free water.
Name of kit and details of any modifications	E	RNeasy micro kit (74004, Qiagen)

**Source of additional reagents used**	**D**	**RNAprotect (76506, Qiagen)**
**Details of DNase or RNase treatment**	**E**	**DNase I treatment (RNeasy micro kit, 74004, Qiagen)**
**Contamination assessment (DNA or RNA)**	**E**	**Reverse transcription without enzyme were performed in order to assess the absence of DNA in the RNA sample.**
**Nucleic acid quantification**	**E**	**RNA concentration was determined by measuring the absorbance at 260 nm UV light**
**Instrument and method**	**E**	**Nanodrop 2000 spectrophotometer (ThermoFisher Scientific)**
**Purity (A260/A280)**	**D**	**RNA purity was determined by measuring the absorbance ratio 260/280. Only samples with purity A260/A280 ≥ 1.8 were used in reverse transcription-quantitative PCR.**
**Yield**	**D**	**Typical yields were 200-600 ng of RNA per eyecup and 0.5-2 µg per retina.**
**RNA integrity: method/instrument**	**E**	**Not performed**
**RIN/RQI or Cq 3' and 5' transcripts**	**E**	**Not performed**
**Electrophoresis traces**	**D**	**Not performed**
**Inhibition testing (Cq dilutions, spike or other)**	**E**	**Cq dilution**
**Reverse transcription**		
**Complete reaction conditions**	**E**	**Total RNA was reverse-transcribed in a final volume of 20 µL with 200 units of Superscript III Reverse Transcriptase (18080-044, ThermoFisher Scientific), 250 ng of random hexamers and 150 ng or 500 ng of RNA for RPE and retina samples, respectively. For primer annealing, 10 µl of total RNA solution was incubated with 1 µL of random hexamers and 1 µL of dNTP mix. Then, 8 µL of RT mix were added: 4 µL of 5X Buffer, 1 µL of 0.1 M DTT, 1 µL of reverse transcriptase and 2 µL RNase-free water. Reverse transcription reactions were performed in a thermal cycler (Eppendorf). cDNA were then diluted 1/5 with RNAse-free water before storage.**
**Amount of RNA and reaction volume**	**E**	**RNA amount: 150 ng total RNA for RPE samples, 500 ng of total RNA for retina samples. RT final volume: 20 µL.**
**Priming oligonucleotide (if using GSP) and concentration**	**E**	**250 ng of random hexamers per RT reaction**
**Reverse transcriptase and concentration**	**E**	**Superscript III RT (200 units/µL)**

Temperature and time	E	Primer annealing: 65°C for 5 minutes and then incubated on ice for 5 minutes. RT reaction: 25°C for 5 minutes, 50°C for 60 minutes and 70°C for 15 minutes
Manufacturer of reagents and catalogue numbers	D	Random hexamers (48190-011, Invitrogen) dNTP 10mM (R0192, ThermoFisher Scientific), Superscript III RT (18080-044, Invitrogen)
Cqs with and without reverse transcription	D	No amplification was detected (i.e. fluorescence remained below detection threshold) in reactions without RT.
Storage conditions of cDNA	D	-20°C
qPCR target information		
Gene symbol	E	See Table S1
Sequence accession number	E	See Table S1
Location of amplicon	D	Not included
Amplicon length	E	See Table S1
In silico specificity screen (BLAST, and so on)	E	Screened using BLAST
Pseudogenes, retropseudogenes, or other homologs?	D	None
Sequence alignment	D	100% alignment between primers and targeted sequence.
Secondary structure analysis of amplicon	D	Not determined
Location of each primer by exon or intron (if applicable)	E	See Table S1
What splice variants are targeted?	E	See Table S1
qPCR oligonucleotides		
Primer sequences	E	See Table S1
RTPrimerDB identification number	D	Not applicable
Probe sequences	D	Not applicable
Location and identity of any modifications	E	No modifications were done
Manufacturer of oligonucleotides	D	IDT oligo

**Purification method**	**D**	**Desalted**
**qPCR protocol**		
**Complete reaction conditions**	**E**	**Reactions contain 250 nM of each primer, 10 µL qPCR mix, and 5 µL of cDNA in a total volume of 20 µL.**
**Reaction volume and amount of cDNA/DNA**	**E**	**Reaction volume: 20 µL. Amount of cDNA: 5 µL of RT diluted 1/5**
**Primer, (probe), Mg2+, and dNTP concentrations**	**E**	**250 nM of each primer, SYBR Green PCR Master Mix contains Mg^{2+} and dNTP**
**Polymerase identity and concentration**	**E**	**AmpliTaq Gold® DNA Polymerase**
**Buffer/kit identity and manufacturer**	**E**	**SYBR Green PCR Master Mix (Applied Biosystems)**
**Exact chemical composition of the buffer**	**D**	**Unknown**
**Additives (SYBR Green I, DMSO, and so forth)**	**E**	**SYBR Green 1 Dye is present in the mix**
**Manufacturer of plates/tubes and catalogue number**	**D**	**MicroAmp Optical 96 Well Reaction Plate (4306737, Applied Biosystems)**
**Complete thermocycling parameters**	**E**	**50°C for 2 minutes, 95°C for 10 minutes, then 40 cycles of 95°C for 15 seconds and 60°C for 1 minute**
**Reaction set up (manual/robotic)**	**D**	**Manual**
**Manufacturer of qPCR instrument**	**D**	**7900HT Fast real-time PCR system (ThermoFisher Scientific)**
**qPCR validation**		
**Evidence of optimization (from gradients)**	**D**	**Primers were designed to have a Tm of 62°C and all qPCR were performed using the same annealing temperature.**
**Specificity (gel, sequence, melt, or digest)**	**E**	**For primers testing, gene-specific amplification was confirmed by a single band of expected size in 2% agarose gel electrophoresis. Melting curve analysis was performed on every reaction at the end of the qPCR run.**
**For SYBR green I, Cq of the NTC**	**E**	**No amplification was detected (i.e. fluorescence remained below detection threshold) in NTC reactions.**
**Calibration curves with slope and y intercept**	**E**	**Calibration curves were generated using a 10-fold dilution series of 7 different concentrations of a standard (PCR fragment of a known copy number). Each calibration curve was measured in duplicates and present Cq plotted against the logarithm of input amount of standard material.**

		**Hprt: y = -3.5722 x + 40.811**<br><br>**Tfeb: y = -3.523 x + 42.327**<br><br>**Mapllc3a: y = -3.465 x +39.38**<br><br>**Atp6v0a: y = -3.2704 x +42.6**<br><br>**Ctsd: y = -3.3185 x+ 31.552**<br><br>**Ccl2: y = -3.5459 x +39.293**<br><br>**Ccl5: y = -3.4362 x +39.474**<br><br>**Cryaa: y = -3.3152 x +42.459**<br><br>**Cryab: y = -3.4448 x +40.16**<br><br>**Cryba1: y = -3.4589 x +41.219**<br><br>**Cryba4: y = -3.5542 x +38.515**<br><br>**Crybb2: y = -3.336 x +43.042**<br><br>**Cryga: y = -3.4528 x +41.589**<br><br>**Crygs: y = -3.611 x +40.16**<br><br>**Fabp5: y = -3.445 x +40.386**
**PCR efficiency calculated from slope**	**E**	**PCR efficiency (E) was calculated from the calibration curve slope using the following formula: $E = (10^{-1/slope} - 1) \times 100$**<br><br>**Hprt:91% Tfeb:92% Mapllc3a:94% Atp6v0a:102% Ctsd:100% Ccl2:91% Ccl5:95% Cryaa:100% Cryab:95% Cryba1:95% Cryba4:91% Crybb2:99% Cryga:95% Crygs:89% Fabp5:95%**
**Confidence intervals for PCR efficiency or SE**	**D**	**Standard error**
**r^2 of calibration curve**	**E**	**Hprt:0.9861 Tfeb:0.9952 Mapllc3a:0.9976 Atp6v0a:0.9878 Ctsd:0.9981 Ccl2:0.9903 Ccl5:0.9813 Cryaa:0.9962 Cryab:0.9877 Cryba1:0.9869 Cryba4:0.9875 Crybb2:0.9599 Cryga:0.9866 Crygs:0.9926 Fabp5:0.9791**
**Linear dynamic range**	**E**	**Linear dynamic range was established for 10^3 to 10^8 copies per reaction.**
**Cq variation at limit of detection**	**E**	**Not determined**
**Confidence intervals throughout range**	**D**	**Not determined**

**Evidence for limit of detection**	**E**	**Not determined**
**If multiplex, efficiency and limit of detection of each assay**	**E**	**Not applicable**
**Data analysis**		
**qPCR analysis program (source, version)**	**E**	**SDS 2.3 and RQ manager 1.2 (Applied Biosystems)**
**Method of Cq determination**	**E**	**A threshold was manually applied to the amplification plots in RQ manager software in order to calculate the Cq values.**
**Outliner identification and disposition**	**E**	**Reactions with abnormal melting curves and large variation between technical replicates were discarded.**
**Results for NTCs**	**E**	**Fluorescence remained below detection threshold in NTC reactions.**
**Justification of number and choice of reference genes**	**E**	**See Liu et al. Gene 2016;580:41-46.**
**Description of normalization method**	**E**	**Using the relative standard curve method, we determined the copy number of target and reference genes by interpolating values from the calibration curves.**
**Number and concordance of biological replicates**	**D**	**At least 5 biological replicates were used (*n* = 5 to 10). Concordance is presented as standard errors (SEM) on graphs.**
**Number of stage (reverse transcription or qPCR) of technical replicates**	**E**	**qPCR reactions were performed in triplicates.**
**Repeatability (intra-assay variation)**	**E**	**Triplicates had Cq standard deviation < 0.3**
**Reproducibility (inter-assay variation, CV)**	**D**	**Not determined. All experiments were done in one laboratory.**
**Power analysis**	**D**	**Not determined**
**Statistical methods for results significance**	**E**	**Student's *t*-test or Welch's *t*-test**
**Software (source, version)**	**E**	**GraphPad Prism 7**
**Cq or raw data submission with RDML**	**D**	**Not done**

Checklist from Bustin et al. (Clinical Chemistry 2009;55:4 611-622), showing (E) essential and (D) desirable information to be included in research reports using qPCR.
